# PCNA-Dependent Cleavage and Degradation of SDE2 Regulates Response to Replication Stress

**DOI:** 10.1371/journal.pgen.1006465

**Published:** 2016-12-01

**Authors:** Ukhyun Jo, Winson Cai, Jingming Wang, Yoojin Kwon, Alan D. D’Andrea, Hyungjin Kim

**Affiliations:** 1 Department of Pharmacological Sciences, Stony Brook University, Stony Brook, New York, United States of America; 2 Department of Radiation Oncology and Center for DNA damage and Repair, Dana-Farber Cancer Institute, Boston, Massachusetts, United States of America; Cancer Research UK/Medical Research Council Oxford Institute for Radiation Oncology, UNITED KINGDOM

## Abstract

Maintaining genomic integrity during DNA replication is essential for cellular survival and for preventing tumorigenesis. Proliferating cell nuclear antigen (PCNA) functions as a processivity factor for DNA replication, and posttranslational modification of PCNA plays a key role in coordinating DNA repair against replication-blocking lesions by providing a platform to recruit factors required for DNA repair and cell cycle control. Here, we identify human SDE2 as a new genome surveillance factor regulated by PCNA interaction. SDE2 contains an N-terminal ubiquitin-like (UBL) fold, which is cleaved at a diglycine motif via a PCNA-interacting peptide (PIP) box and deubiquitinating enzyme activity. The cleaved SDE2 is required for negatively regulating ultraviolet damage-inducible PCNA monoubiquitination and counteracting replication stress. The cleaved SDE2 products need to be degraded by the CRL4^CDT2^ ubiquitin E3 ligase in a cell cycle- and DNA damage-dependent manner, and failure to degrade SDE2 impairs S phase progression and cellular survival. Collectively, this study uncovers a new role for CRL4^CDT2^ in protecting genomic integrity against replication stress via regulated proteolysis of PCNA-associated SDE2 and provides insights into how an integrated UBL domain within linear polypeptide sequence controls protein stability and function.

## Author Summary

Preserving genomic integrity during DNA replication is essential for preventing tumorigenesis. The CRL4^CDT2^ ubiquitin E3 ligase plays a unique role in this pathway by coupling proteolysis to interaction with the DNA replication processivity factor PCNA, in order to ensure selective elimination of key factors in cell cycle regulation. However, the mechanisms by which CRL4^CDT2^ directly regulates replication-associated DNA repair remain elusive. In this work, we identify a new human protein called SDE2 that helps cells relieve replication stress and ensure completing DNA replication process, whose activity is regulated by PCNA interaction and CRL4^CDT2^. We show that SDE2 is cleaved by PCNA interaction and ubiquitin signaling to generate a functional C-terminal product. The cleaved SDE2 negatively regulates PCNA monoubiquitination required for relieving replication stress. Conversely, the cleaved SDE2 fragments need to be degraded, and failure to degrade SDE2 impairs S phase progression and cellular survival. Our findings uncover the role of CRL4^CDT2^-proteolytic signaling coupled to PCNA in protecting genomic integrity against replication stress. Knowledge on such mechanism will be useful to identify novel cancer therapeutic interventions exploiting deregulated ubiquitin signaling and SDE2 activities in cancer.

## Introduction

Replication stress caused by aberrant DNA replication is a major source of genome instability [1]. Consequently, DNA mutations and chromosome aberrations arising from stalled and collapsed replication forks are closely associated with tumorigenesis [2]. To counteract these genotoxic threats, cells make a concerted effort to activate cell cycle checkpoints and execute DNA repair, processes that are collectively referred to as the DNA damage response (DDR) [3]. Because many DNA lesions block replication fork progression in S phase, they need to be repaired or bypassed to complete DNA replication in time. Posttranslational modification of the DNA replication processivity factor Proliferating cell nuclear antigen (PCNA) plays a key role in coordinating this process by providing a scaffold to recruit factors required for DNA damage tolerance mechanisms to replicate over a lesion that blocks replicative polymerase progression, which includes translesion DNA synthesis (TLS) or template switching. In particular, PCNA monoubiquitination triggers TLS to directly bypass base damage and temporarily relieve arrested DNA replication using low-fidelity TLS polymerases that can accommodate various types of template structures [4]. In this mechanism, single-stranded (ss) DNA generated at stalled replication forks activates RAD18 ubiquitin E3 ligase to monoubiquitinate PCNA [5,6]. Subsequently, TLS polymerases are recruited to monoubiquitinated PCNA (PCNA-Ub) via both a specialized ubiquitin-binding domain that recognizes the monoubiquitin moiety of PCNA and a PCNA-interacting protein (PIP) box motif [7–9]. The PIP box binds to a hydrophobic pocket of PCNA buried under the interdomain-connecting loop, and interaction among PIP box-containing proteins can be competitive [10,11]. Multiple pathways are required for regulating PCNA-mediated TLS steps, including RAD18 recruitment, PCNA deubiquitination, TLS polymerase extraction, and chromatin remodeling [12–16]. Importantly, deregulation of PCNA deubiquitination caused by a deficiency of the deubiquitinating enzyme (DUB) ubiquitin-specific protease 1 (USP1) leads to disruption of TLS and increased genome instability due to the spurious recruitment of TLS polymerases [17].

Another important feature of PCNA-controlled DNA replication is proteolysis coupled to DNA synthesis and repair. PCNA interaction via the PIP box regulates the degradation of specific protein targets, which is required for proper cell cycle progression [18]. The best example is PCNA-dependent degradation of the licensing factor CDT1 [19]. To ensure single origin licensing per cell cycle, CDT1 in the pre-replication complex (pre-RC) is degraded by the CRL4^CDT2^ ubiquitin E3 ligase complex after recruiting the MCM2-7 complex to replication origins in G1 phase [20,21]. Interestingly, CRL4^CDT2^ only recognizes PCNA-associated CDT1, which represents the CDT1 population coupled to DNA replication [22]. The PIP box in the N-terminus of CDT1 functions as a degron that specifically recruits CDT2 to its target CDT1 only when it is bound to PCNA [23]. DNA is also required for CDT1 degradation, indicating that PCNA^DNA^-PIP degron-CRL4^CDT2^ constitutes a ternary complex that targets substrates for destruction. In addition to CDT1, other PIP degron-containing proteins such as SET8 and p21 are degraded by CRL4^CDT2^ in S phase to prevent premature chromatin compaction and to relieve inhibition of PCNA, respectively, thus highlighting the role of CRL4^CDT2^-dependent proteolysis in ensuring DNA replication [24–26]. These CRL4^CDT2^ substrates are also degraded following DNA damage, suggesting that they must also be properly removed to facilitate DNA repair.

Proteolysis is mediated by the covalent conjugation of polyubiquitin chains to a substrate, which is in turn targeted to the 26S proteasome for destruction [27]. This process is reversible, and removal of ubiquitin from a substrate is mediated by DUBs. DUBs cleave the isopeptide bond between the C-terminal diglycine residues of ubiquitin and the amino group of a lysine in a substrate [28]. Most DUBs are cysteine proteases that rely on the thiol group of a cysteine in the active site [29]. DUBs antagonize the activity of ubiquitin E3 ligases, thereby fine-tuning the stability and activity of target proteins.

In contrast to ubiquitin and ubiquitin-like modifiers covalently attached to target proteins via a lysine side chain, some proteins bear a ubiquitin-like (UBL) motif within their linear polypeptide sequence [30]. These so-called ubiquitin-domain proteins (UDPs) constitute a structurally and functionally diverse protein group that regulates ubiquitin signaling. For instance, Rad23, a protein involved in nucleotide excision repair, contains a UBL domain at its N-terminus, which interacts with the ubiquitin-interacting motif of the 19S proteasome regulatory subunit to deliver substrates for degradation [31,32]. Other variant ubiquitin-regulatory X (UBX) domain-containing proteins such as p47 and the Npl4/Ufd1 complex function as essential cofactors for ATPase p97 to remove misfolded proteins in the ERAD pathway [33,34]. UBL domains also regulate the activities of proteins that contain it. For example, USP1, a DUB that regulates the Fanconi anemia DNA interstrand cross-link repair and TLS, auto-cleaves the C-terminus of the integrated UBL at a diglycine motif via its catalytic activity, resulting in its subsequent degradation by the proteasome [16].

C1orf55 (hereinafter referred to as SDE2) is a highly conserved human protein that contains an SDE2 domain of unknown function and a DNA-binding SAP (SAF-A/B, Acinus and PIAS) domain (S1A Fig). SAP domains are frequently found in proteins participating in the DNA repair process, such as PIAS1, Ku70, and RAD18 [35] (S1B Fig). SDE2 is also a target of ATM/ATR kinases, further suggesting its possible function in the DDR or in DNA repair [36]. Sde2 in *S*. *pombe* is known to be involved in genome maintenance pathway, but its function in higher eukaryotes remains uncharacterized [37]. Here, we identify that the UBL domain of SDE2 plays an important role in regulating replication stress response. The N-terminal UBL has a PIP box that mediates its interaction with PCNA, which leads to the cleavage of SDE2 at a diglycine motif and subsequent degradation by CRL4^CDT2^. The cleaved C-terminal SDE2 is required for antagonizing PCNA monoubiquitination triggered by ultraviolet (UV) damage. By contrast, failure to degrade SDE2, due to its defective cleavage, impedes S phase progression and cellular survival. We propose that CRL4^CDT2^, a known regulator of the cell cycle and DNA repair, counteracts replication stress by balancing the activity of PCNA-associated SDE2 at replication forks, using ubiquitin-like fold of SDE2 as a signal for targeted cleavage and degradation.

## Results

### SDE2 releases an N-terminal ubiquitin-like domain cleaved at a conserved diglycine motif

**Fig 1:**
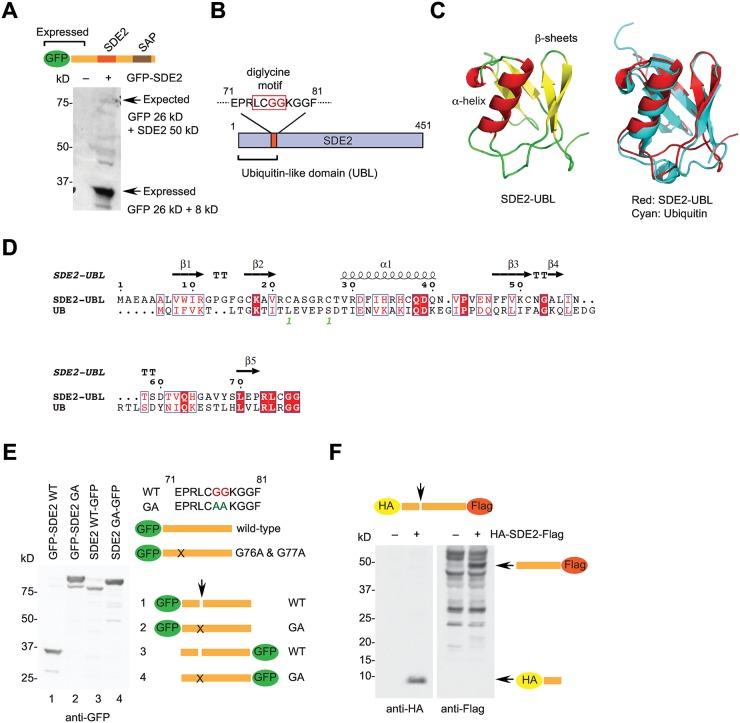
SDE2 is cleaved at a conserved diglycine motif to release an N-terminal UBL. **(A)** HeLa cells transfected with N-terminal GFP-tagged SDE2 cDNA were analyzed by Western blotting. **(B)** A schematic showing a diglycine motif (red box) that resembles the C-terminus of ubiquitin. **(C)** A model structure of the SDE2-UBL predicted by Phyre2, superimposed with ubiquitin (PDB: 1D3Z) on the right. **(D)** Structure-based sequence alignment of the SDE2-UBL and ubiquitin generated by ESPript. **(E)** Confirmation of SDE2 cleavage. HeLa cells transfected with the indicated GFP-tagged plasmids were analyzed by Western blotting. **(F)** Cleavage of HA-SDE2-Flag visualized by anti-HA and anti-Flag Western blotting from duplicated HeLa cell lysates.

To investigate the function of SDE2, we first cloned and expressed an N-terminal GFP-tagged full-length human SDE2 in HeLa cells, which is expected to migrate as an approximately 76 kD band in SDS-PAGE. However, we only observed a band below 37 kD (Fig 1A). Because we did not find any mutations that may have caused accidental truncation, we reasoned that SDE2 may be cleaved close to its N-terminus to generate an 8 kD polypeptide fused to GFP. Analysis of the SDE2 amino acid sequence revealed conserved diglycine residues at Gly76 & 77 (Fig 1B and S1A Fig). The diglycine motif is found at the C-terminus of ubiquitin or ubiquitin-like modifiers, and DUB-dependent cleavage releases single ubiquitin-like molecule or processes poly-ubiquitin precursors. Interestingly, the N-terminal 77 amino acid polypeptide of SDE2 exhibits a structural fold almost identical to ubiquitin that consists of an α-helix and several β-sheet folds, as revealed by homology-based structure prediction (Fig 1C and 1D), suggesting that it constitutes a UBL domain. To confirm that SDE2 is cleaved at the diglycine motif, we mutated two glycine residues to alanines (GA) and examined the cleavage pattern of SDE2. In contrast to the wild-type protein, the N-terminal GFP-tagged GA mutant was not cleaved and present as full-length protein (Fig 1E; compare lanes 1 & 2). Comparison of the immunoblot bands for the C-terminally GFP labeled wild-type and GA SDE2 mutants further confirmed that the N-terminal UBL is cleaved at positions 76 and 77 (Fig 1E; lanes 3 & 4). Furthermore, immunoblots of the SDE2 protein that contains both N-terminal HA- and C-terminal Flag-tags showed separation of SDE2 into the two fragments of the predicted lengths (Fig 1F). Using an antibody raised against SDE2, we also confirmed that the endogenous SDE2 protein exists as a fully processed form (S1C Fig). Taken together, these data show that proteolytic cleavage of the N-terminal SDE2 at the site of a signature diglycine motif releases a UBL via DUB-like activity.

### The SDE2-UBL contains a PIP box required for the cleavage of SDE2

**Fig 2:**
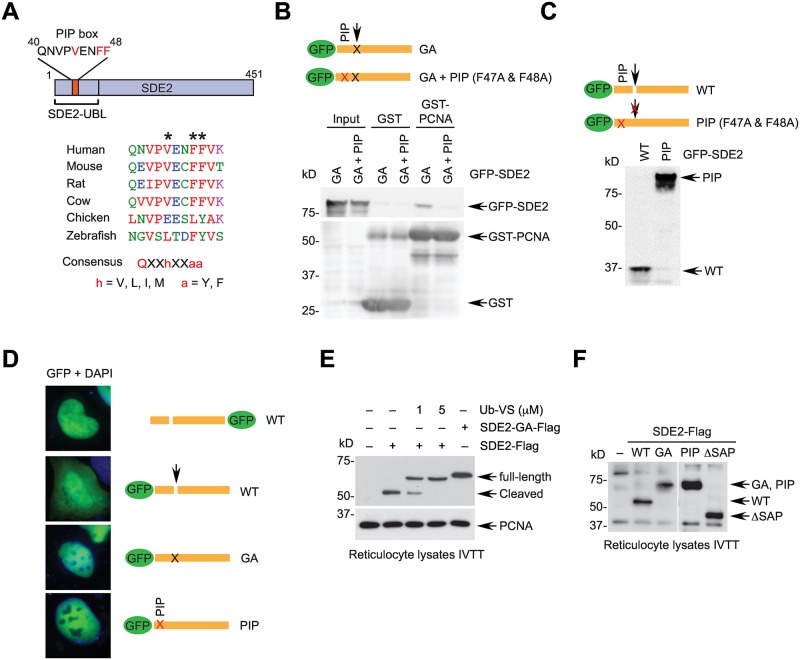
A PIP box in the SDE2-UBL is required for the cleavage of SDE2. **(A)** A schematic showing a PIP box in the SDE2-UBL. PIP boxes from different species are aligned, and consensus elements are marked with asterisks. **(B)** 293T cell lysates expressing GFP-SDE2 GA or GA + PIP (F47A & F48A) mutants were incubated with GST- or GST-PCNA-bound glutathione beads and analyzed by Western blotting. **(C)** HeLa cells expressing GFP-tagged SDE2 wild-type or PIP mutant were analyzed by Western blotting. **(D)** GFP-SDE2 variants transfected into U2OS cells were fixed and visualized by fluorescence microscopy. **(E)** SDE2-Flag proteins *in vitro* transcribed and translated (IVTT) from reticulocyte lysates were analyzed by Western blotting. Where indicated, ubiquitin vinyl sulfone (Ub-VS) was added during expression. **(F)** Inhibition of cleavage of IVTT SDE2-Flag GA and PIP mutants. ΔSAP: aa395-450 deletion.

Although the cleaved N-terminus of SDE2 (SDE2-UBL) exhibits a fold similar to that of ubiquitin, its primary amino acid composition is not identical. Therefore, we further analyzed the SDE2 sequence to gain insights into the role of UBL domain. Interestingly, the UBL contains a conserved PIP box, which resembles those frequently found in Y-family TLS polymerases, lacking the Q residue found at position 1 in canonical PIP boxes (Fig 2A and S2A Fig) [38]. To investigate the role of the PIP box in PCNA interaction and SDE2 cleavage, we examined the interaction between SDE2 and PCNA by GST pull-down. Full-length SDE2 (GA mutant) and the UBL domain of SDE2 interacted with GST-PCNA, whereas the PIP box mutant (F47A & F48A) did not, showing that SDE2 interacts with PCNA and an intact PIP box is required for this interaction (Fig 2B and S2B Fig). Remarkably, when the PIP box mutation was introduced into wild-type SDE2, the GFP-fusion protein was not cleaved at the diglycine motif in cells, suggesting that the PCNA interaction is required for the cleavage of SDE2 (Fig 2C). We further confirmed our observation by visualizing GFP-SDE2 proteins with fluorescence microscopy. SDE2 was primarily present in the nucleus as determined by C-terminally GFP-tagged SDE2, while N-terminal GFP-SDE2 diffused throughout the cell, representing cleaved GFP-UBL (Fig 2D). By contrast, GFP signals of the N-terminal GFP-SDE2 GA or PIP mutants were concentrated in the nucleus. Collectively, these data show that the cleavage of SDE2 is coupled to its interaction with PCNA via the PIP box located at the UBL.

Since a diglycine motif of ubiquitin is recognized by a DUB to be cleaved from its substrate, we next sought to determine whether DUB activity is involved in SDE2 cleavage. When SDE2 was *in vitro* transcribed and translated using a reticulocyte-derived cell free expression system, where PCNA is detected, we observed that SDE2 was fully cleaved upon expression, whereas ubiquitin vinyl sulfone, or ubiquitin aldehyde, which are pan-DUB inhibitors, prevented this process (Fig 2E and S2C Fig). This result indicates that DUB activity in the expression lysate is responsible for cleaving SDE2. Mutation of the diglycine motif or the PIP box, but not the SAP DNA binding domain, abolished the cleavage, suggesting that the interaction with PCNA stimulates DUB activity required for SDE2 cleavage (Fig 2F). Indeed, recombinant SDE2 expressed in *E*. *coli*, which contains a β sliding clamp instead of PCNA in eukaryotes, retained its full-length form (S2D Fig). Given its high efficiency of cleavage, SDE2 might function as a DUB to cleave itself. However, mutations in the conserved Cys (that constitutes an active site for cysteine protease) or His-Glu (for metalloprotease) near the SDE2 domain failed to prevent its cleavage, suggesting that SDE2 does not exhibit intrinsic catalytic activity (S2E Fig).

### The SDE2-UBL is degraded via CRL4^CDT2^-mediated proteolysis

PCNA-mediated cleavage of SDE2 is analogous to PCNA-dependent destruction of CDT1 by CRL4^CDT2^, which is coupled to DNA replication and DNA repair. This process requires a modified PIP box called a PIP degron, which consists of a TD motif at positions 5 and 6 and a basic residue at position +4 (S3A Fig) [18]. The TD motif enhances substrate binding to PCNA, whereas the B+4 residue helps in the recruitment of CDT2, although a non-canonical PIP degron has been reported [18,39,40]. Although the PIP box of SDE2 lacks the features of a canonical PIP degron, requirement of PCNA for the cleavage of SDE2 raises a possibility that CRL4^CDT2^ may be involved in regulating the function of SDE2.

**Fig 3:**
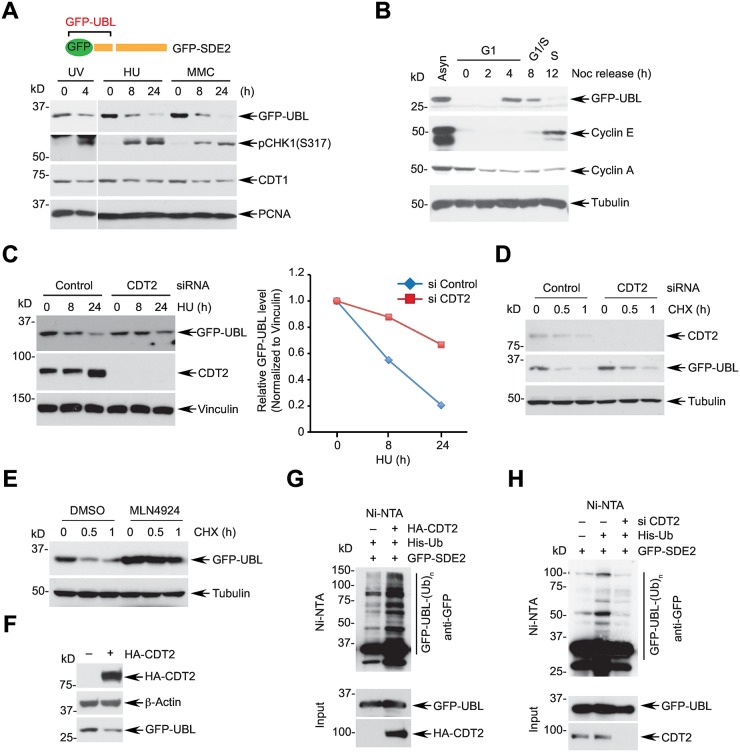
SDE2-UBL undergoes CRL4^CDT2^-dependent proteolysis. **(A)** HeLa cells expressing full-length GFP-SDE2 were left untreated or treated with 40 J/m^2^ ultraviolet C (UVC), 2 mM hydroxyurea (HU), or 1 μM mitomycin C (MMC) for the indicated times, and N-terminal GFP-UBL levels were analyzed by Western blotting. **(B)** HeLa cells exponentially growing or synchronized by nocodazole were collected at the indicated times following mitotic shake-off and analyzed by Western blotting. Cell cycle progression was analyzed by flow cytometry in S3C Fig. **(C)** (left) Full-length GFP-SDE2 expressing HeLa cells transfected with siRNA CDT2 (vs. control) were treated with 2 mM HU for the indicated times and analyzed by Western blotting. (right) Quantification of immunoblots by Image J. **(D)** HeLa cells expressing full-length GFP-SDE2 were transfected with siRNA control or CDT2 for 48 h and treated with 50 μg/mL cycloheximide (CHX) for the indicated times. The level of GFP-UBL was analyzed by Western blotting. **(E)** HeLa cells treated with DMSO or 1 μM MLN4924 were treated with 50 μg/mL CHX for the indicated times, and GFP-UBL levels were analyzed by Western blotting. **(F)** HeLa cells expressing GFP-SDE2 were transfected with HA-CDT2 plasmid (vs. empty vector), and GFP-UBL levels were analyzed by Western blotting. **(G)** 293T cells transfected with the indicated plasmids were treated with 10 μM MG132 for 4 h, lysed in a denaturing condition, and incubated with Ni-NTA agarose for the *in vivo* ubiquitination assay. **(H)** 293T cells transfected with indicated siRNAs and plasmids were subjected to the *in vivo* ubiquitination assay.

Interestingly, treatment with DNA-damaging agents that cause replication stress led to degradation of GFP-UBL that was generated from full-length GFP-SDE2, and proteasome activity was required for its degradation (Fig 3A and S3B Fig). Moreover, cellular levels of GFP-UBL increased at early S phase and then disappeared during S phase progression, similar to other known CDT2 substrates (Fig 3B and S3C Fig). Notably, CDT2 knockdown prevented the degradation of GFP-UBL following HU treatment (Fig 3C). Also, CDT2 depletion slowed down the kinetics of GFP-UBL degradation without damage as measured by cycloheximide blocking (Fig 3D). Furthermore, MLN4924, a small molecule inhibitor of the NEDD8 activating enzyme (NAE) that inhibits CRL4^CDT2^ activity [41], increased basal levels of GFP-UBL and inhibited degradation of GFP-UBL in the absence and presence of DNA damage (Fig 3E and S3D Fig). Additionally, G1 phase cells with CDT2 knockdown restored GFP-UBL levels (S3E Fig). These data show that CRL4^CDT2^ is required for the degradation of SDE2-UBL in a cell cycle- and DNA damage-dependent manner. Conversely, overexpression of CDT2 was sufficient to decrease GFP-UBL levels (Fig 3F), and was able to enhance polyubiquitination of GFP-UBL *in vivo* (Fig 3G). By contrast, CDT2 knockdown led to decreased levels of GFP-UBL polyubiquitin conjugates (Fig 3H). Taken together, these data suggest that CRL4^CDT2^-dependent proteolytic pathway regulates the degradation of SDE2-UBL generated from cleavage.

### The cleavage of SDE2 is prerequisite for degradation of C-terminal SDE2 in chromatin and is regulated by CRL4^CDT2^

**Fig 4:**
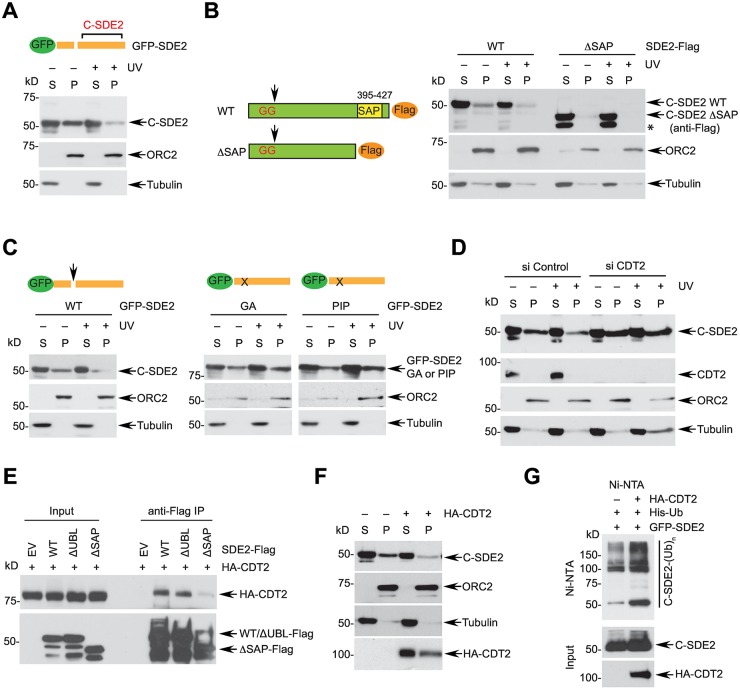
Degradation of cleaved C-terminal SDE2 is regulated by chromatin association and CRL4^CDT2^. **(A)** HeLa cells expressing full-length GFP-SDE2 were left untreated or treated with 40 J/m^2^ UVC for 4 h and fractionated into cytosolic/nucleoplasmic (S) vs. chromatin-enriched (P) fractions. The level of cleaved C-terminal SDE2 (C-SDE2) was analyzed by Western blotting. **(B)** (left) A schematic of the C-terminal Flag-tagged SDE2 ΔSAP mutant (aa 1–394). (right) HeLa cells expressing SDE2-Flag wild-type or ΔSAP mutant were treated with 40 J/m^2^ UVC for 4 h, fractionated, and analyzed by Western blotting. **(C)** HeLa cells expressing full-length GFP-SDE2 variants were irradiated with UVC and fractionated for Western blot analysis. **(D)** HeLa cells transfected with siRNA control or CDT2 for 48 h were treated with 40 J/m^2^ UVC for 4 h and fractionated. Endogenous cleaved SDE2 levels were analyzed by Western blotting. **(E)** 293T cells were transiently expressed with HA-CDT2 along with Flag-SDE2 variants, treated with 10 μM MG132 for 4h, and cell lysates were subjected to anti-Flag IP and Western blotting. **(F)** HeLa cells transfected with HA-CDT2 plasmid were fractionated to S and P fractions, and endogenous C-SDE2 levels were analyzed by Western blotting. **(G)** Immunoblots of the *in vivo* ubiquitination assay in Fig 3G were reprobed to visualize the polyubiquitin conjugates of C-SDE2.

Because degradation of N-terminal UBL is mediated by CRL4^CDT2^, we next expressed full-length GFP-SDE2 and examined whether degradation of C-terminal SDE2 (C-SDE2) upon cleavage is subjected to similar regulation. When cells were fractionated using cytoskeleton (CSK) buffer, we observed that C-SDE2 was specifically degraded in the chromatin-enriched fraction following UVC irradiation, which was antagonized by proteasome inhibition (Fig 4A and S4A Fig). The level of C-SDE2 also exhibited a cell cycle-dependent change in chromatin, which showed transient accumulation at G1/S transition followed by gradual decrease in S phase (S4B Fig). Since a SAP domain is known to mediate the interaction with DNA, we determined if the SAP domain of SDE2 is required for SDE2 degradation. Deletion of the SAP DNA binding domain did not affect the cleavage of SDE2 but abrogated the association of C-SDE2 in the chromatin fraction, therefore the SAP mutant failed to undergo degradation following UVC irradiation (Fig 4B). The half-life of the SAP mutant also increased in the absence of damage (S4C Fig). We observed a further processed form of the cleaved SAP mutant, whose identity is currently unknown (Fig 4B, asterisk). Importantly, both non-cleavable SDE2 GA and PIP mutants failed to undergo degradation in chromatin following UVC irradiation (Fig 4C), and the half-life of the GA mutant greatly increased (S4D Fig), indicating that SDE2 needs to be cleaved for subsequent degradation as a normal turnover process and in response to DNA damage.

Interestingly, similarly to SDE2-UBL, knockdown of CDT2 rescued endogenous C-SDE2 levels in chromatin that were decreased by UVC irradiation and during the cell cycle (Fig 4D and S4E Fig), and a decreased amount of C-SDE2 polyubiquitin conjugates was observed (S4F Fig). Although CDT2 depletion is known to arrest cells in G2 phase, we observed that cells progressed proficiently through S phase as shown by increase in BrdU incorporation (S4G Fig), and C-SDE2 degradation of cells traversing to G1 from G2 arrest was abolished by CDT2 knockdown, indicating that the phenotype is not an indirect effect of S phase defect or G2 arrest (S4H Fig). Furthermore, as is the case with SDE2-UBL, MLN4924 abolished UVC-induced C-SDE2 degradation in chromatin (S4I Fig). Therefore, we reasoned that degradation of C-SDE2 could also be regulated by CRL4^CDT2^ similarly to its N-terminus. Although C-SDE2 itself does not contain a PIP degron, anti-Flag immunoprecipitation revealed that CDT2 interacts with C-terminally Flag-tagged SDE2, and exogenously expressed C-SDE2 without its UBL retains its interaction, indicating that C-SDE2 alone is sufficient to interact with CDT2 (Fig 4E). Notably, deletion of the SAP domain abolished the interaction of C-SDE2 with CDT2, suggesting that the C-terminal region of SDE2 may contain an uncharacterized CDT2 degron motif different from a canonical PIP degron. Furthermore, the interaction between C-SDE2 and CDT2 was retained in the presence of Benzonase endonuclease, indicating that the interaction is direct (S4J Fig). Accordingly, overexpression of CDT2 reduced C-SDE2 levels in chromatin and enhanced polyubiquitin conjugates of C-SDE2 (Fig 4F and 4G). Taken together, these data indicate that CRL4^CDT2^ is an important regulator that degrades both N-terminal UBL and C-terminal SDE2 products once generated by a DUB-dependent cleavage of SDE2. Given the role of the SAP domain in localizing SDE2 in chromatin, we do not exclude the possibility of DNA in contributing or strengthening the formation of the PCNA^DNA^-SDE2-CRL4^CDT2^ ternary complex.

### SDE2 prevents DNA damage arising from replication stress

**Fig 5:**
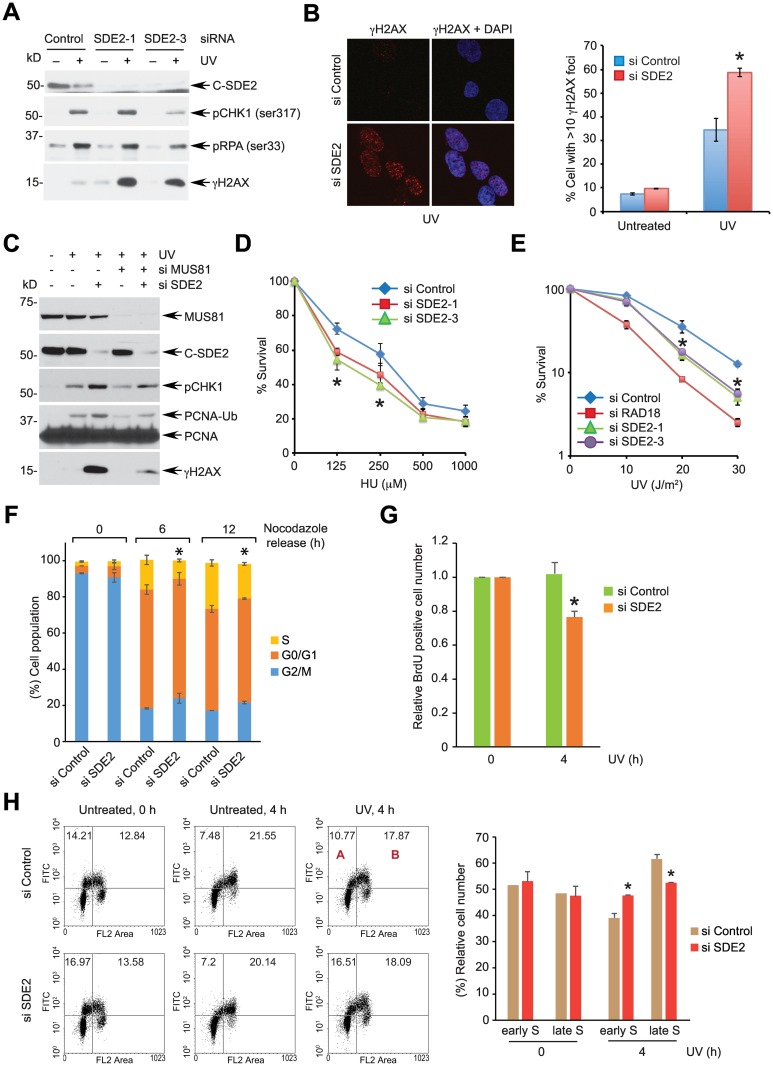
SDE2 depletion causes replication-associated DNA damage and a defect in replication stress response. **(A)** SDE2 knockdown leads to increased levels of γH2AX upon DNA damage. HeLa cells transfected with the indicated siRNAs were treated with 40 J/m^2^ UVC for 4 h and analyzed by Western blotting. **(B)** (left) siRNA-transfected U2OS cells were treated with 40 J/m^2^ UVC for 4 h, and γH2AX foci were analyzed by immunofluorescence. (right) Quantification of cells displaying more than 10 γH2AX foci. Data shown are the mean ± SD from three independent experiments. * *p* < 0.01 compared with siRNA control. **(C)** MUS81 depletion suppresses damage-induced γH2AX caused by SDE2 knockdown. HeLa cells transfected with indicated siRNA oligoes were treated with 40 J/m^2^ for 4 h, and cell lysates were analyzed by Western blotting. Note that PCNA monoubiquitination was decreased upon MUS81 knockdown (related to Fig 7). **(D, E)** Luminescence-based viability (D) or clonogenic survival (E) of siRNA-transfected HeLa cells treated with the indicated doses of DNA damage. Data shown are the mean ± SD from three independent experiments. * *p* < 0.01 SDE2 knockdown compared with control (except 250 μM HU *p* < 0.05). **(F)** SDE2 knockdown causes a defect in S phase progression. HeLa cells transfected with siRNA control or SDE2 were synchronized at G2/M phase by treating 100 ng/mL nocodazole for 16 h. After mitotic shake-off, cells were released into G1 and S phases, and cell cycle was monitored by PI staining and flow cytometry. Data shown are the mean ± SD from three independent experiments. * *p* < 0.05 for S phase population from cells with SDE2 knockdown vs. control. **(G)** HeLa cells transfected with siRNA control or SDE2 were left untreated or treated with 40 J/m^2^ UVC, and incubated with 10 μM BrdU for 0.5 h before harvest at 4 h post UVC irradiation. S phase cells were determined by anti-BrdU/PI staining and flow cytometry, and SDE2-depleted BrdU^+^ cells were normalized by control-treated BrdU^+^ cells. Data shown are the mean ± SD from two independent experiments. * *p* < 0.01 SDE2 knockdown vs. control. **(H)** Decreased replication recovery of SDE2-depleted cells against UV damage. HeLa cells transfected with siRNA control or SDE2 were pulsed with 10 μM BrdU for 0.5 h, left untreated or treated with 40 J/m^2^ UVC, and released into fresh medium for 4 h. (left) Representative cell cycle distribution measured by anti-BrdU/PI staining and flow cytometry. (right) Relative distribution of early S (A/A+B) and late S (B/A+B) cells out of total BrdU^+^ cells. Data shown are the mean ± SD from three independent experiments. * *p* < 0.01 for increased early and decreased late S populations from cells with SDE2 knockdown vs. control.

Many CRL4^CDT2^ substrates regulate cell cycle and DNA repair [42]. To investigate the possible role of SDE2 in regulating cell cycle progression and the DDR, we depleted SDE2 levels using multiple independent siRNA oligos (S5A Fig). Depletion of SDE2 in HeLa cells led to significantly elevated γH2AX levels, but not phosphorylated CHK1 or RPA, following UVC irradiation as revealed by Western blotting and immunofluorescence, representing increased DNA damage in the absence of SDE2 (Fig 5A and 5B, and S5B Fig). A persistent stalled replication fork leads to replication collapse, ultimately leading to double-strand breaks (DSBs). Activity of MUS81, a structure-specific nuclease, has been implicated in the processing of stalled forks [43]. Indeed, enhanced γH2AX signal resulting from SDE2 knockdown could be reduced by co-depleting MUS81, indicating that SDE2 depletion results in the formation of replication-associated DSBs (Fig 5C). Accordingly, cells depleted of SDE2 were hypersensitive to replication-associated DNA damage caused by UVC, HU, or aphidicolin (Fig 5D and 5E, and S5C Fig). By contrast, SDE2-depleted cells were not sensitive to poly(ADP-ribose) polymerase (PARP) inhibition, suggesting that homologous recombination is not directly affected by SDE2 knockdown (S5D Fig). Also, we did not observe significant difference in cellular resistance to MMC and camptothecin in the absence of SDE2, indicating that SDE2 may be involved in a confined and specific DNA repair pathway, which is often the case with distinct DNA repair factors (S5E and S5F Fig).

Although knockdown of SDE2 itself did not affect overall cell cycle distribution in the absence of damage (S5G Fig), SDE2-depleted cells traversed more slowly from G1 to S phase when they were synchronized and released from nocodozale compared to control cells, arguing for a defect in S phase progression (Fig 5F). To further substantiate this finding, we assessed the ability of SDE2 to promote recovery from replication fork stalling by visualizing S phase progression following UVC irradiation. When we measured BrdU-positive cell population following UVC irradiation, SDE2-depleted cells exhibited decreased S phase cells in comparison to control cells that showed minimal reduction of cycling cells, indicating that replication recovery following UV damage is impaired (Fig 5G and S5H Fig). In addition, when we pulse-labeled cells with BrdU and followed labeled cells through S phase, we observed that loss of SDE2 led to a significant decrease of percentage in late S population with a concomitant increase in early S population, arguing for slower S phase progression in the presence of UV damage (Fig 5H). Thus, SDE2 antagonizes replication-associated DNA damage by allowing efficient replication fork recovery and completion of DNA replication when cells are exposed to replication stress.

### Degradation of SDE2 is required for S phase progression and cellular survival

**Fig 6:**
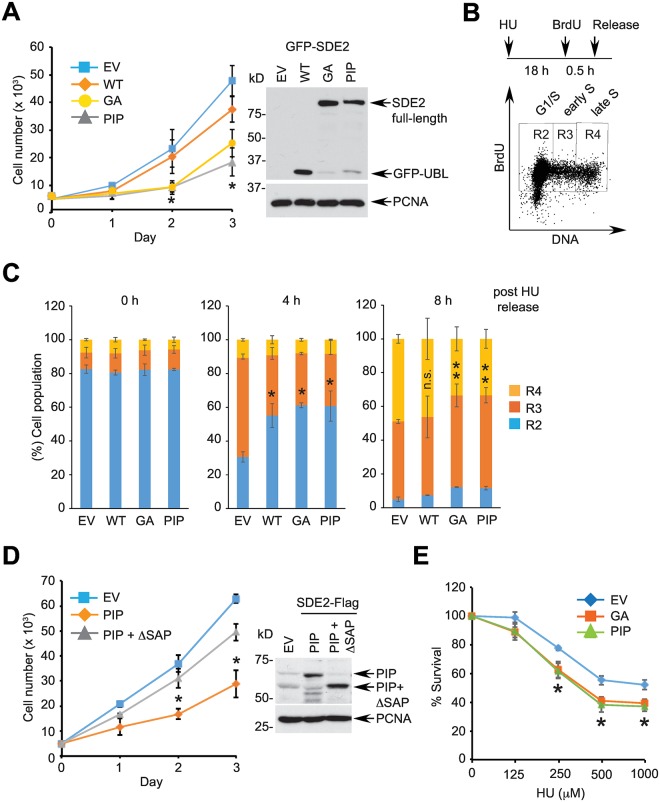
Failure to degrade SDE2 impairs S phase progression and cellular survival. **(A)** (left) A total of 5 x 10^3^ HeLa cells transiently expressing GFP-SDE2 variants were plated, and cell numbers were counted at the indicated times. Data shown are the mean ± SD of three independent experiments. * *p* < 0.01 GA and PIP compared with vector or wild-type. (top) The expression of SDE2 variants was analyzed by Western blotting. (EV: empty vector) **(B)** A schematic for monitoring S phase progression. HeLa cells were incubated with 2 mM HU for 18 h, pulsed with 10 μM BrdU for 30 min, and released into fresh medium. A representative flow cytometry gating of BrdU^+^ cells that traverse from G1/S boundary (R2) to early (R3) and late (R4) S phases is shown. **(C)** HeLa cells expressing vector control, wild-type, GA, or PIP SDE2-Flag variants were analyzed as (B) following release from HU. Data shown are the mean ± SD of two independent experiments. * *p* < 0.01 for R2 cells compared with EV. ** *p* < 0.01 R3 cells compared with EV. n.s.: not significant compared with EV. **(D)** (left) A total of 5 x 10^3^ HeLa cells stably expressing vector, SDE2-Flag PIP or PIP with ΔSAP mutant were plated, and cell numbers were counted at the indicated times. Data shown are the mean ± SD of three independent experiments. * *p* < 0.01 compared with empty vector or PIP. (right) Expression of SDE2 variants was analyzed by Western blotting. **(E)** HeLa cells transiently expressing GFP-SDE2 variants were plated to 96 well plates, treated with indicated doses of HU, and cell viability was determined by CellTiter-Glo luminescence. Data shown are the mean ± SD of three independent experiments. * *p* < 0.01 for GA and PIP compared to empty vector.

CDT2 has been shown to degrade its substrates including p21, SET8, and FBH1 during S phase progression or following DNA damage to limit their potential negative effects on DNA replication and repair [42]. Degradation of C-SDE2 during S phase progression and after DNA damage suggests that SDE2 must also be properly removed. This would be required for preventing accumulation of SDE2 at DNA lesions near replication forks, which could be detrimental to cells. Therefore, we determined whether enforced expression of non-cleavable SDE2 mutants that cannot be degraded exerts any negative effect on counteracting replication stress. When wild-type SDE2 was overexpressed in HeLa cells, it marginally reduced cellular proliferation. By contrast, overexpression of SDE2 GA or PIP mutants led to a significant delay of cell doublings, indicating that aberrant accumulation of SDE2 impedes cellular proliferation (Fig 6A). We next assessed the ability of these cells to progress through S phase following replication stress. HeLa cells synchronized at the G1/S transition by HU were pulse-labeled with BrdU, and progression into S phase was monitored (Fig 6B). When compared to vector control, cells expressing wild-type SDE2 exhibited a transient delay in progressing from early to late S phase, whereas cells expressing GA or PIP SDE2 mutants exhibited persistently delayed S phase progression (Fig 6C; compare 4 h vs. 8 h and S6A Fig). These data indicate that improper clearance of SDE2 and subsequent accumulation interferes with S phase progression. Importantly, deletion of the SAP domain abolished the effect of the PIP mutant in impairing cellular proliferation, suggesting that aberrant occupancy of SDE2 at DNA via the SAP domain impedes cell growth (Fig 6D). Accordingly, cells expressing GA or PIP SDE2 mutants were hypersensitive to HU treatment, supporting the notion that a defect in SDE2 turnover is detrimental to cellular survival (Fig 6E). Exogenous expression of the GA mutant itself did not cause elevated γH2AX levels in contrast to SDE2 deletion upon UVC damage, suggesting that mechanisms whereby loss-of-function or gain-of-function of SDE2 impact cellular survival may be distinct (S6B Fig) Taken together, these results indicate that SDE2 needs to undergo timely degradation following cleavage to allow for cell cycle progression and recovery from DNA damage for ensuring cell survival.

### SDE2 negatively regulates PCNA monoubiquitination required for TLS

**Fig 7:**
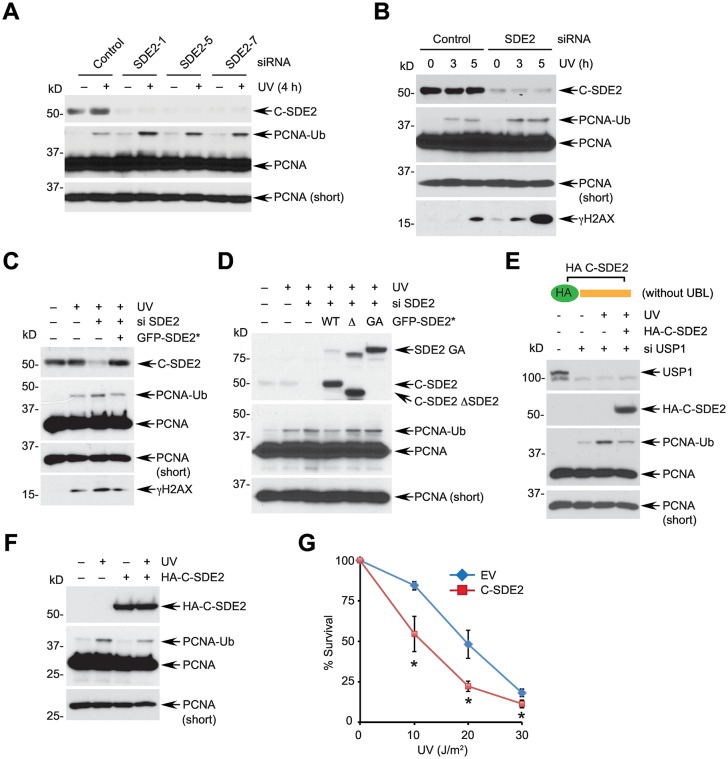
SDE2 suppresses UVC-inducible PCNA monoubiquitination. **(A-B)** Western blot analyses of siRNA-transfected HeLa cells irradiated with 40 J/m^2^ UVC for the indicated times. **(C)** HeLa cells were sequentially transfected with SDE2 siRNA and siRNA-resistant GFP-SDE2 cDNA (GFP-SDE2*), UVC irradiated, and analyzed by Western blotting. **(D)** HeLa cells transfected with SDE2 siRNA were transiently expressed with siRNA-resistant GFP-SDE2 wild-type, ΔSDE2, or GA mutant, UVC irradiated, and analyzed by Western blotting. **(E)** Expression of cleaved C-terminal SDE2 is sufficient to suppress the increased PCNA-Ub levels caused by USP1 depletion. HeLa cells were sequentially transfected with USP1 siRNA (vs. control) and HA-C-SDE2 encoding plasmid (vs. empty vector) and analyzed by Western blotting. **(F)** HeLa cells expressing vector or HA-C-SDE2 were treated with 40 J/m^2^ UVC for 4 h, and PCNA-Ub levels were analyzed by Western blotting. **(G)** Cells in (F) were plated in 6 well plates, irradiated UVC, and cellular viability was determined by clonogenic survival at 12 days. Data shown are the mean ± SD of three independent experiments. * *p* < 0.01 compared with empty vector.

Given that SDE2 is regulated by PCNA association and counteracts DNA damage in response to replication stress, we next determined whether SDE2 is involved in regulating the DNA damage tolerance pathway that helps cells deal with replication-blocking DNA lesions. USP1 is responsible for reversible deubiquitination of PCNA-Ub and thereby controls TLS [16]. Accordingly, deficiency of USP1 leads to elevation of PCNA-Ub levels and disrupts TLS regulation [44]. Interestingly, we observed that depletion of SDE2 using multiple independent siRNAs resulted in increased levels of PCNA-Ub in HeLa and U2OS cells (Fig 7A and S7A Fig). SDE2 knockdown increased the formation of UVC-induced PCNA-Ub in a time- and dose-dependent manner (Fig 7B and S7B Fig). Conversely, CDT2 knockdown disrupted the turnover of SDE2 and resulted in reduced PCNA-Ub, indicating that increased SDE2 can antagonize the PCNA modification process (S7C Fig). Importantly, damage-induced PCNA-Ub levels were reverted to normal levels by introduction of siRNA-resistant full-length SDE2 (Fig 7C). However, expression of GA or the SDE2 domain-deletion mutant failed to decrease PCNA-Ub levels, suggesting that the SDE2 domain of cleaved SDE2 is essential for suppressing PCNA monoubiquitination (Fig 7D). Notably, exogenous expression of HA-tagged C-SDE2 was able to antagonize the elevated PCNA-Ub levels caused by USP1 knockdown and UVC damage, suggesting that activated SDE2 regulates PCNA modification process independently of USP1 (Fig 7E). Moreover, RAD18 depletion could decrease γH2AX levels elevated by SDE2 knockdown besides negating formation of PCNA-Ub following UVC damage, indicating that deregulation of PCNA-Ub levels could be one source of heightened replication-associated DNA damage caused by SDE2 depletion (S7D Fig).

Interestingly, exogenously expressed HA-tagged C-SDE2 was not properly degraded in comparison to full-length SDE2 and maintained its high levels (S7E Fig). Deregulation of PCNA monoubiquitination caused by RAD18 or USP1 deficiency leads to impaired cellular survival against UVC damage [44,45]. Similarly, elevated exogenous levels of C-SDE2 were sufficient to decrease UVC-induced PCNA-Ub, and cells became hypersensitive to UVC irradiation (Fig 7F and 7G). Collectively, our results implicate for the role of SDE2 in negatively regulating PCNA monoubiquitination, thereby contributing to the fine-tuning of PCNA modification during replication stress. We speculate that deregulation of SDE2 proteolysis compromises DNA damage tolerance and prevents efficient recovery of stalled replication forks, leading to decreased cellular viability.

## Discussion

### SDE2: A new player required for preserving genomic integrity

**Fig 8:**
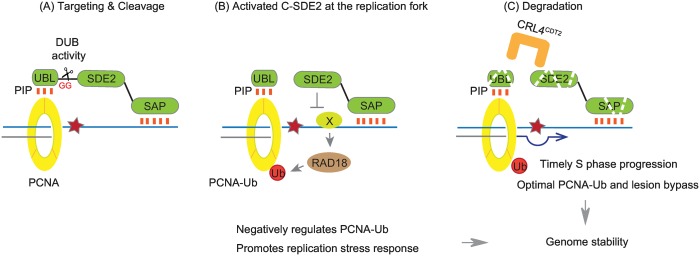
A proposed model for the regulation of SDE2 by PCNA-dependent cleavage and degradation. **(A)** Targeting of SDE2 to PCNA-associated replication forks via the N-terminal UBL containing a PIP box leads to the cleavage of SDE2 at the diglycine motif. DUB activity is required for its cleavage. **(B)** The cleaved C-terminal SDE2 functions as a negative regulator of damage-inducible RAD18-dependent PCNA monoubiquitination. The SDE2 domain is required for this process. **(C)** Degradation of the cleaved N-terminal and C-terminal SDE2 products by CRL4^CDT2^ allows timely S phase progression and promotes replication stress response, at least partly through PCNA-Ub-dependent lesion bypass, to ensure genome stability. Deregulation of SDE2 levels, either by knockdown or by defective proteolysis, disrupts this genome maintenance pathway.

In this study, we identify human SDE2 as a new factor required for counteracting replication stress. PCNA-dependent processing of SDE2 generates a functional protein that negatively regulates damage-inducible PCNA monoubiquitination, which in turn needs to be eliminated by proteolysis to allow for S phase progression and replication fork recovery in response to DNA damage (Fig 8). Once cleaved, SDE2 may be required for restricting unscheduled PCNA modification before DNA replication or fine-tune monoubiquitination process in the context of replication stress. Accordingly, SDE2 depletion leads to elevated replication-associated DNA damage and impaired cellular survival. By contrast, prolonged accumulation of SDE2, due to a defect in cleavage or degradation, is expected to impede S phase progression, at least partly resulting from disruption of the balanced levels of damage-inducible PCNA ubiquitination. Similar phenotype of the GA and PIP mutants also suggests that aberrant accumulation of unprocessed SDE2 at DNA, presumably at replication forks via its SAP DNA binding domain, impedes cell cycle progression and is harmful to cells. Alternatively, the N-terminal UBL domain, if not properly degraded, may directly compete with TLS polymerases for occupying the surface of PCNA. Indeed, PIP-degron-containing peptides have been shown to impair Pol η foci formation [46].

Sde2 in *S*. *pombe* was initially identified in the *sde2*^+^ (silencing defective 2) strain, which shows defective telomere silencing [37]. Yeast Sde2 was proposed to mediate the recruitment of SHREC, a histone deacetylase complex, to telomeres, thereby maintaining heterochromatin status. Interestingly, Sde2 lacks the C-terminal SAP domain and S/TQ ATM/ATR phosphorylation sites (S1A Fig), suggesting that higher eukaryotes have evolved additional functions in the DDR and DNA repair. Currently, our mutation analysis argues against the idea that SDE2 exerts auto-DUB activity or functions as a DUB for PNCA-Ub (S2E Fig). In addition, USP1, a DUB for PCNA-Ub, does not play a role in cleaving SDE2 (S8A Fig). The exact mechanism by which SDE2 regulates PCNA ubiquitination is currently unknown. SDE2 may directly antagonize the activity of signaling proteins or nucleases, whose activity is required for remodeling replication forks and recruiting RAD18 ubiquitin E3 ligase to ssDNA. The SDE2 domain may mediate the interaction with this unknown factor, and loss of its interaction would lead to hyper-activation of RAD18-dependent PCNA monoubiquitination process. Suppression of γH2AX foci and PCNA monoubiquitination by co-depletion of MUS81 with SDE2 supports this possibility. Alternatively, SDE2 may function as an enzyme that directly regulates replication stress response. Failure to counteract replication stress may indirectly elevate PCNA monoubiquitination due to extensive ssDNAs or aberrant fork structures. Notably, SDE2 exhibits domain organization and regulatory principles that are similar to Wss1 and its human homolog DVC1 (SPRTN/C1orf124) DNA-protein cross-link proteases known to participate in DNA damage tolerance and replication stress response [47] (S8B Fig). DVC1 regulates PCNA monoubiquitination and TLS polymerase extraction from PCNA-Ub [12,13,48,49]. The SprT-like metallopeptidase domain of DVC1 is associated with counteracting replication stress, premature aging, and tumorigenesis [50,51]. Targeting of Wss1 to DNA lesions requires DNA and SUMO interactions, whereas DVC1 utilizes the interaction with PCNA and ubiquitin via its PIP box and UBZ4 domain [12,52]. Their activity is further regulated either by self-cleavage (Wss1) or by proteasomal degradation (DVC1) [13,52]. Another Wss1-like protease family, Wlm2, contains an N-terminal UBL upstream of the SprT domain analogous to SDE2 (S8 Fig). Although highly speculative, these observations suggest that the SDE2 domain may have uncharacterized catalytic functions to relieve replication stress at stalled replication forks. The exact mechanism by which SDE2 promotes response to replication stress, and how its deregulation impacts genomic integrity and tumorigenesis are important future directions to pursue.

### PCNA-dependent cleavage and degradation of SDE2 by CRL4^CDT2^

Our study identifies a new substrate of the CRL4^CDT2^ ubiquitin E3 ligase, whose activity is regulated by PCNA that provides a docking site for CDT2. Interestingly, SDE2 cleavage, a prerequisite for degradation, also requires PCNA association, revealing a complex layer of substrate regulation by the PCNA^DNA^-PIP degron-CRL4^CDT2^ ternary complex. The PIP box in the SDE2-UBL is used not only as a degradation signal but also as a targeting element for cleavage. Coupling of the PIP box to the UBL domain is likely to ensure that only the processed (i.e., functional) form is subjected to degradation in chromatin, coordinated by CRL4^CDT2^-mediated proteolysis. Both the N- and C-terminal pieces may be held together by an unknown factor upon cleavage, such that CRL4^CDT2^ directly polyubiquitinates both fragments. A yet-to-be identified ubiquitin E3 ligase may cooperate with CRL4^CDT2^ to degrade C-SDE2, and activity of such an enzyme may be activated by the DDR to regulate damage-dependent C-SDE2 degradation. Unlike known CRL4^CDT2^ substrates, SDE2 does not contain a canonical PIP degron (S3A Fig). For a substrate that lacks a TD motif and a B+4 residue in its PIP box, other motif often compensates for these suboptimal elements. For instance, CDT2-dependent degradation of FBH1 that does not have a TD motif in the N-terminal PIP box is compensated by an APIM motif, another PCNA-interacting element identified in the C-terminal FBH1 [39]. Therefore, a distinct motif can increase the regulatory capacity of PCNA-dependent proteolysis by CRL4^CDT2^, and other regions of SDE2 such as the SAP domain may cooperate with its suboptimal PIP box for CRL4^CDT2^-dependent SDE2 degradation when degradation of two fragments is coordinated. The requirement of the SAP domain for CDT2 interaction and SDE2 turnover supports this idea. Our data also suggest that additional CRL4^CDT2^ substrates could be found that do not present the classical PIP degron consensus.

The mechanism by which PCNA interaction leads to SDE2 cleavage remains unknown. Nearly completed cleavage of exogenously expressed SDE2 indicates that a DUB should be tightly associated to PCNA, and increased local concentration of substrates may aid in the processing of SDE2. PCNA interaction may induce a conformation change of SDE2 that exposes the N-terminal UBL, which is otherwise buried under the rest of SDE2.

### Ubiquitin-like domain: A new class of ubiquitin signaling for regulating protein function

Processing of SDE2 via a diglycine motif provides a remarkable example of how a cleavable ubiquitin-like structural element is utilized to regulate protein localization, stability and function. The presence of a PIP box in the UBL allows SDE2 to target to replication forks and undergo activation by cleavage. Although not perfectly matched, the C-terminal sequence of the SDE2-UBL, _72_PRLCGG_77_, is analogous to ubiquitin _71_LRLRGG_76_, suggesting that recognition of these residues by a DUB is sufficient to induce cleavage along with additional structural information from a ubiquitin-like fold. DUB(s) responsible for cleaving SDE2 are currently unknown. It would be important to identify such DUB(s) to understand how ubiquitin signaling is coupled to PCNA association to regulate DNA replication and genome stability.

Although many proteins have integrated UBL domains, the function of these domains remains largely unexplored. Interestingly, structural bioinformatics analysis revealed that UBL domains are frequently found in the USP DUB family, either inside or outside of their catalytic core [53]. Given the role of conjugated ubiquitin or ubiquitin-like modifiers, including SUMO, Nedd8, and ISG15, in controlling diverse cellular processes, integrated UBL domains are expected to similarly regulate enzyme activity and substrate specificity [54]. Indeed, auto-cleavage of USP1 at its diglycine motif leads to USP1 degradation following DNA damage to potentiate PCNA monoubiquitination [16]. Analogous to UBL, the SUMO-like domain (SLD) of UAF1, the activator of USP1, recruits FANCD2-Ub and PCNA-Ub to USP1 via the interaction between SLD and the SUMO-interacting motif (SIM) of FANCI and ELG1, binding partners of FANCD2 and PCNA, respectively [55]. The growing list of UDPs, as revealed in this study, underscores the diversity of ubiquitin-like signaling in controlling fundamental cellular functions, including the DDR and DNA repair.

## Materials and Methods

### Cell culture and plasmid construction

HeLa, U2OS, and 293T cells were cultured in Dulbecco’s Modified Eagles Medium supplemented with 10% fetal bovine serum following standard culture conditions and procedures. Human *SDE2* and *CDT2* cDNA were acquired from Open Biosystems (MHS1010-97228092) and the Dana-Farber/Harvard Cancer Center DNA Resource Core (HsCD00330875), respectively. The full-length or deleted cDNA was PCR-amplified and subcloned into pcDNA3-Flag, pcDNA3-HA (Invitrogen), pEGFP-C1/N1 (Clontech), and pGEX6P-1 (GE Healthcare Life Sciences). Point mutations were introduced using the QuikChange II XL Site-Directed Mutagenesis Kit (Agilent Technologies) and confirmed by DNA sequencing. Stable cell lines were generated by retroviral transduction of pMSCV-SDE2-Flag constructs, followed by selection in the presence of 2 μg/mL puromycin.

### Transfection and siRNA

Plasmid transfection was performed using GeneJuice (Millipore) according to the manufacturer’s protocols. siRNA duplexes were synthesized by Qiagen or Ambion and were transfected at 25 nM using Lipofectamine RNAiMAX (Invitrogen). The siRNA sequences can be found in S1 Table.

### Antibodies and chemicals

Antibodies used for Western blot analysis included the following: anti-C1orf55/SDE2 (epitope: a.a. 318–410; Sigma-Aldrich), anti-GFP (JL-8, Clontech), anti-HA (6E2, Cell Signaling), anti-pCHK1 (S317, Cell Signaling), anti-Actin (Cell Signaling), anti-CDT1 (Cell Signaling), anti-Flag (M2, Sigma-Aldrich), anti-Tubulin (Sigma-Aldrich), Cyclin E (H-12, Santa Cruz), anti-PCNA (PC-10, Santa Cruz), anti-Vinculin (H-300, Santa Cruz), anti-GST (B-14, Santa Cruz), Cyclin A (H-432, Santa Cruz), anti-γH2AX (JBW301, Millipore), anti-CDT2 (Bethyl), anti-pRPA (S33, Bethyl), pKAP-1 (S824, Bethyl), anti-ORC2 (BD Pharmingen), and anti-MUS81 (MTA30 2G10/3, Abcam). Mitomycin C, camptothecin, hydroxyurea, cycloheximide, aphidicolin, and Z-Leu-Leu-Leu-al (MG132) were purchased from Sigma-Aldrich. Rucaparib (AG-014699) was purchased from Selleckchem. MLN4924, ubiquitin vinyl sulfone, and ubiquitin aldehyde were purchased from Boston Biochem. Drugs were used at the concentrations indicated in the figure legends.

### Western blotting, fractionation, and co-immunoprecipitation

Cells were lysed with NETN300 buffer (1% NP40, 300 mM NaCl, 0.1 mM EDTA, and 50 mM Tris [pH 7.5]) supplemented with protease inhibitor cocktail (Roche), resolved by SDS-PAGE gels and transferred onto PVDF membranes (Millipore), and then antibodies were detected using the enhanced chemiluminescence method (Western Lightening, Perkin Elmer). Subcellular fractionation was performed as previously described [56]. Briefly, cells were incubated with 0.1% Triton X-100 cytoskeleton (CSK) buffer (10 mM Tris [pH 6.8], 100 mM NaCl, 300 mM sucrose, MgCl_2_, 1 mM EGTA, 1 mM EDTA, and 0.1% Triton X-100) for 5 min on ice. After centrifugation at 1500 g for 5 min, the supernatant (S) was separated from the pellet (P), and pellets were sequentially resolved in PBS and 2X boiling lysis buffer (50 mM Tris-Cl [pH 6.8], 2% SDS, and 850 mM β-mercaptoethanol), which was followed by 10 min of boiling. For anti-Flag co-immunoprecipitation, cells were lysed with NETN150 buffer (1% NP40, 150 mM NaCl, 0.1 mM EDTA, and 50 mM Tris [pH 7.5]) supplemented with protease inhibitor cocktail and centrifuged at 15,000 rpm for 10 min. Supernatants were incubated with anti-Flag M2 affinity gel (Sigma) and the immune complexes were washed with NETN150 buffer for four times. Where indicated, 10 U of Benzonase Nuclease (EMD Millipore) was added during the lysis to degrade DNA and RNA.

### BrdU staining and flow cytometry

Cells were incubated with 10 μM BrdU (Sigma-Aldrich) for 30 min before harvest following DNA damage or cell cycle synchronization. Cells were fixed with 70% ethanol at 4°C overnight and incubated with 2 N HCl/0.5% Triton X-100 for 30 min at RT. After being washed with 0.1 M sodium tetraborate and PBS-T (PBS + 0.5% BSA + 0.2% Tween 20), cell pellets were incubated with 1:100 anti-BrdU (BU-1, Thermo Fisher) and 1:1,000 Alexa Fluor^®^ 568 goat anti-mouse IgG (Molecular Probes) for 30 min each at RT. Cells were washed with PBS and resuspended with 10 μg/mL RNase A and 20 μg/mL propidium iodide (Sigma-Aldrich). Cell cycle profiles were analyzed using a FACS Caliber (BD Bioscience).

### RT-qPCR

RNA was isolated using TRIzol (Invitrogen). cDNA synthesis was performed using a high-capacity cDNA reverse transcription kit (Applied Biosystems) according to the manufacturer’s protocols. The mRNA levels were quantified using a Fast SYBR Green Master Mix (Applied Biosystems) and StepOnePlus Real-time PCR system (Applied Biosystems). *GAPDH* mRNA was used as an internal control.

### Cell cycle synchronization

HeLa cells were incubated with 2 mM thymidine (Sigma-Aldrich) for 24 h, released into fresh medium for 3 h, and incubated with 100 ng/mL nocodazole (Sigma-Aldrich) for 12 h. Cells in the G2/M phase were collected by mitotic shake-off and released into fresh medium.

### Fluorescence microscopy

For GFP fluorescence, cells were grown on coverslips, UVC irradiated, fixed with 4% paraformaldehyde for 10 min at RT, washed three times with PBS and mounted with DAPI-containing mounting medium (Vector Lab). For γH2AX foci, fixed cells were permeabilized with PBS/ 0.3% Triton X-100 and blocked with 2% bovine serum albumin (BSA, Sigma-Aldrich). Cells were incubated with an anti-γH2AX primary antibody (1: 500, Millipore) in PBS/ 1% BSA and 1:1000 Alexa Fluor^®^ 568 goat anti-mouse IgG secondary antibody (Molecular Probes). Images were captured using a Zeiss LSM 510 inverted confocal microscope with Zeiss LSM software.

### *In vivo* ubiquitin assay

*In vivo* ubiquitin assays were performed in denaturing conditions. 293T cells that were treated with 10 μM MG132 for 4 h were resuspended with PBS/1% SDS, snap-frozen in liquid nitrogen, and boiled for 15 min. Cell lysates were diluted 10 times with PBS and centrifuged at 15,000 rpm for 15 min at 4°C. Lysates (4%) were saved for input, and lysates were incubated with HisPur Ni-NTA Resin (Thermo Fisher) in the presence of 10 mM imidazole (Sigma-Aldrich) at 4°C for 3 h, followed by five washes with PBS/0.1% SDS, 10 mM imidazole. Resins were boiled in 2X Laemmli sample buffer and subjected to SDS-PAGE.

### *In vitro* transcription and translation

A total of 125 ng of pcDNA3 plasmids were incubated with 5 μL of TnT^®^ T7 Quick Coupled Transcription/Translation Master Mix (Promega) at 30°C for 70 min, and 1 μL aliquots were analyzed by Western blotting. For inhibiting DUB activity, HA-tagged ubiquitin vinyl sulfone or ubiquitin aldehyde (Boston Biochem) were added to the reaction mixture along with the plasmids.

### Protein purification and GST pull-down assay

GST pull-down was performed as previously described [15]. For the interaction between GST-PCNA and SDE2, GST or GST-PCNA was expressed in BL21 (DE3) strains using 0.5 mM Isopropyl β-D-1-thiogalactopyranoside (IPTG, Sigma-Aldrich) at 30°C. Cells were lysed in PBS with lysozyme, sonicated, and further incubated with 1% Triton X-100. Cell lysates were recovered by centrifugation at 15,000 rpm at 4°C for 15 min and incubated with glutathione-sepharose beads (GE Healthcare). After washing, the beads were incubated with 293T cell lysates in NETN150 buffer (1% NP40, 150 mM NaCl, 0.1 mM EDTA, and 50 mM Tris [pH 7.5]) supplemented with protease inhibitor cocktail (Roche) for 3 h at 4°C followed by three washes. For SDE2 expression, full-length SDE2 cDNA was cloned into the pGEX6P-1 vector. Expression in *E*. *coli* BL21 (DE3) was induced by 0.5 mM IPTG at 30°C for 6 h during exponential growth, purified with glutathione-sepharose beads, and visualized by Coomassie staining.

### Cell viability assay

For clonogenic survival, siRNA-treated cells were seeded on the 6-well dishes at a density of 1,000 cells per well and irradiated with increasing doses of UVC at 48 h after transfection. Colonies were stained after 12 days using Crystal Violet (0.5%) in methanol and counted. For other types of DNA damage, siRNA-transfected cells were seeded on the 96-well plates and treated with individual DNA damaging agents in duplicate at 48 h after transfection. Cell viability was determined using the CellTiter-Glo luminescent cell viability assay (Promega) 5 days after continuous drug treatment. Luminescence was measured using a Centro LB960 Microplate Luminometer (Berthold Technologies) and Mikrowin 2000 software.

### Bioinformatics analysis

The sequence alignment was performed using Clustal Omega. Structure modeling was performed using Phyre2 to predict the 3D structure of the SDE2-UBL, with the crystal structure of ubiquitin (PDB: 1D3Z) used as a template. PyMoL was used to superimpose the 3D structures [57]. The structure-based sequence alignment between SDE2-UBL and ubiquitin was presented using ESPript3 [58].

### Statistical analysis

*P* values for statistical analyses were obtained using Student’s *t* test.

## Supporting Information

S1 Table List of siRNA sequences. (DOCX)

S1 Fig Structure of SDE2 (Related to Fig 1). **(A)** A sequence alignment of SDE2 from different species. The conserved diglycine motif is marked in a box. **(B)** A sequence alignment of the SDE2 SAP domain with known SAP domains. The SAP domain consists of two bipartite α-helices enriched with hydrophobic amino acids (i.e., Leu, Val; indicated with black asterisks), which are separated by an invariant glycine residue (red asterisk). A positively charged amino acid such as lysine (light blue asterisk) is expected to make contact with the backbone of DNA. The alignment was performed using Clustal Omega and presented using Jalview. **(C)** Endogenous SDE2 is processed to release its UBL. To determine the size of full-length and cleaved SDE2, cell lysates expressing C-terminal Flag-tagged SDE2 wild-type or GA mutants were analyzed by Western blotting with anti-Flag and anti-SDE2 antibodies. The epitope of SDE2 antibody falls within amino acids 318–410. Only fully processed endogenous SDE2 is detected (compare lanes 1 and 3). * denotes nonspecific bands.
(TIF)

S2 Fig Interaction of SDE2 with PCNA (Related to Fig 2). **(A)** Analysis of the SDE2 PIP box. Both canonical and non-canonical PIP boxes from several known PIP-box-containing proteins are presented, and conserved elements are marked in red. **(B)** Interaction of GFP-SDE2-UBL with PCNA. 293T cell lysates expressing GFP-SDE2-UBL wild-type or PIP mutant (F47A & F48A) were incubated with GST- or GST-PCNA-bound glutathione beads and analyzed by Western blotting. **(C)** SDE2-Flag proteins *in vitro* transcribed and translated (IVTT) from reticulocyte lysates were analyzed by Western blotting. Where indicated, 5 μM ubiquitin aldehyde (Ub-Al) was added during expression. **(D)** Expression of full-length GST-tagged SDE2. GST-SDE2 was induced from the *E*. *coli* BL21 strain by 0.5 mM IPTG at 30°C. Proteins were captured with glutathione-conjugated beads and visualized by Coomassie staining. **(E)** Conserved cysteine or histidine-glutamate residues are not required for SDE2 cleavage. The indicated SDE2-Flag wild-type or point mutants were *in vitro* transcribed and translated, and cleaved SDE2-Flag proteins were analyzed by Western blotting.
(TIF)

S3 Fig Degradation of SDE2-UBL (Related to Fig 3). **(A)** Sequence alignment of PIP degron motifs present in known CDT2 substrates. Canonical PIP residues are shown in red, and PIP degron-specific residues are shown in blue. Several substrates lack elements constituting a classical PIP degron. **(B)** DNA-damage dependent degradation of SDE2-UBL is mediated by the proteasome. HeLa cells expressing GFP-SDE2 were left untreated (Unt) or treated with 40 J/m^2^ ultraviolet C (UVC) for 4 h, 2 mM hydroxyurea (HU) for 8 h, and 1 μM mitomycin C (MMC) for 16 h, and cellular GFP-UBL levels were analyzed by Western blotting. Where indicated, cells were treated with 10 μM MG132 for 4 h before harvest. **(C)** Cell cycle profiles of synchronized HeLa cells in Fig 3B determined by flow cytometry **(D)** HeLa cells expressing full-length GFP-SDE2 was treated with 1 μM MLN4924 and irradiated with 40 J/m^2^ UVC for 4 h. The GFP-UBL levels were analyzed by Western blotting. **(E)** GFP-SDE2-expressing HeLa cells transfected with siRNA control or CDT2 were synchronized by 100 ng/mL nocodazole at the G2/M phase and released for 2 h. The GFP-UBL levels were analyzed by Western blotting.
(TIF)

S4 Fig The elements required for degradation of C-SDE2 (Related to Fig 4). **(A)** Degradation of C-SDE2 is proteasome-dependent. HeLa cells were left untreated or treated with 40 J/m^2^ UVC for 4 h, fractionated into cytosolic/nucleoplasmic (S) and chromatin-enriched (P) fractions using CSK buffer, and the endogenous C-SDE2 levels were analyzed by Western blotting. Where indicated, cells were treated with 10 μM MG132 for 4 h before harvest. **(B)** C-SDE2 levels are regulated in a cell cycle-dependent manner. HeLa cells were synchronized with nocodazole for 12 h and released into fresh medium after mitotic shake-off. Cells were harvested at the indicated times, and endogenous C-SDE2 levels were analyzed by Western blotting. The cell-cycle dependent change of C-SDE2 association in chromatin is quantified by ImageJ and indicated below the blots. **(C, D)** The half-life of C-SDE2 is extended by ΔSAP or GA mutations. (top) HeLa cells expressing full-length SDE2-Flag wild-type or mutants were with 50 μg/mL of CHX, and cell lysates were analyzed by Western blotting. (bottom) Quantification of immunoblots by Image J. The dotted line indicates a half-life. **(E)** CDT2 is required for the degradation of C-SDE2 during cell cycle progression. HeLa cells transfected with siRNA control or CDT2 were synchronized with nocodazole and released, and cell lysates were analyzed by Western blotting to check endogenous C-SDE2. **(F)** CDT2 is required for polyubiquitination of C-SDE2. Immunoblots of the *in vivo* ubiquitination assay in Fig 3H were reprobed with anti-SDE2 antibody to check the polyubiquitin conjugates of C-SDE2 in the absence of CDT2. **(G)** Cell cycle profile and BrdU incorporation of siRNA-transfected cells were analyzed by PI staining or 30 min BrdU incubation followed by flow cytometry, respectively. **(H)** siRNA-transfected cells were synchronized by G2/M phase by 100 ng/mL nocodazole, treated with 40 J/m^2^ UVC, and released into G1 after mitotic shake-off. Degradation of C-SDE2 in chromatin was analyzed by fractionation and Western blotting. **(I)** Inhibition of CRL4^CDT2^ activity prevents SDE2 degradation in chromatin following DNA damage. HeLa cells were treated with 1 μM MLN4924 (vs. DMSO) for 24 h, treated with 40 J/m^2^ UVC for 4 h, and fractionated for Western blot analysis. **(J)** The Co-IP experiment was performed as Fig 4E in the presence of 10 U Benzonase, and the immune complex was analyzed by Western blotting.
(TIF)

S5 Fig SDE2 is required for counteracting replication stress (Related to Fig 5). **(A)** Quantification of *SDE2* mRNA by RT-qPCR following transfection of SDE2 siRNA for 48 h. Data shown are the mean ± SD from three independent experiments. * *p* < 0.01 compared with siRNA control. **(B)** siRNA-transfected U2OS cells were analyzed by Western blotting to confirm the elevated γH2AX levels following UVC irradiation. (**C-F**) Luminescence-based viability assay of HeLa cells transfected with the indicated siRNAs for 48 h. Cell viability was determined by CellTiter-Glo 5 days after treatment with the indicated doses of aphidicolin (C), rucaparib (PARP inhibitor; D), MMC (E), or camptothecin (F). **(G)** Cell cycle distribution of HeLa cells transfected with siRNA control or SDE2 analyzed by PI staining and flow cytometry. **(H)** Representative flow cytometry of cells in Fig 5H following 40 J/m^2^ UVC irradiation and 0.5 h BrdU pulse before harvest at 4 h post treatment. The percentage of BrdU^+^ cells were gated and labeled.
(TIF)

S6 Fig Failure to degrade SDE2 impairs S phase progression (Related to Fig 6). **(A)** A representative set of flow cytometry panels showing delayed S progression of the SDE2 GA and PIP mutants following release from HU treatment. (B) HeLa cells transfected with siRNA SDE2 or plasmid encoding the C-terminally Flag-tagged SDE2 GA mutant were irradiated with 40 J/m^2^ UVC for 4 h and analyzed by Western blotting.
(TIF)

S7 Fig Negative regulation of PCNA monoubiquitination by SDE2 (Related to Fig 7). **(A)** Knockdown of SDE2 leads to increased PCNA monoubiquitination induced by UVC irradiation. U2OS cells transfected with the indicated siRNAs were irradiated with 40 J/m^2^ UVC for 4 h, and cell lysates were analyzed by Western blotting. **(B)** Knockdown of SDE2 increases PCNA monoubiquitination in a dose-dependent manner. siRNA-transfected HeLa cells were irradiated with the indicated doses of UVC and analyzed by Western blotting. **(C)** Depletion of CDT2, which prevents UVC-inducible SDE2 degradation, leads to decreased PCNA monoubiquitination. HeLa cells transfected with the indicated siRNAs were irradiated with 40 J/m^2^ UVC for 4 h and fractionated with CSK buffer for Western blot analysis. **(D)** siRNA-transfected HeLa cells were treated with 40 J/m^2^ UVC for 4 h, and γH2AX and PCNA-Ub levels were analyzed by Western blotting. **(E)** HeLa cells expressing wild-type SDE2-Flag or HA-tagged C-SDE2 were treated with 50 μg/mL of CHX, and cell lysates were analyzed by Western blotting.
(TIF)

S8 Fig Structural comparison of several SprT-like metalloprotease family proteins and SDE2 (Related to discussion). **(A)** HeLa cells were transfected with siRNA control or USP1, and endogenous SDE2 was analyzed by Western blotting. **(B)** Human DVC1 (DNA-damage protein targeting VCP), yeast Wss1 (weak suppressor of smt3), and yeast Wlm2 (Wss1-like metalloproteases) share several functional motifs including the SprT-like metalloprotease motif, Cdc48/p97-interacting motif (SHP-box and VIM), and ubiquitin- (UBZ), SUMO- (SIM) binding motif. DVC1 has a PIP box that allows for targeting to PCNA along with a UBZ motif. The N-terminus of yeast Wlm2 constitutes a ubiquitin-like domain similar to that of SDE2. hsDVC1 (*Homo Sapiens* NP_114407); ssWss1 (*Saccharomyces Cerevisiae* NP_012002.1); spWlm2 (*Saccharomyces Pombe* NP_588321.1); spSde2 (*Saccharomyces Pombe* NP_594019.1); hsSDE2 (*Homo Sapiens* NP_689821).
(TIF)
