# Supplementary material for: PCNA-Dependent Cleavage and Degradation of SDE2 Regulates Response to Replication Stress
Source: PLoS Genet. 2016 Dec 1;12(12):e1006465. doi: 10.1371/journal.pgen.1006465 (PMC5131917; doi:10.1371/journal.pgen.1006465)
Supplement: S1 Table — (DOCX) [file pgen.1006465.s001.docx]

| **Target** | **Sequence (5′-3′)** | **Manufacturer** |
| --- | --- | --- |
| Control | GGGTATCGACGATTACAAATT | Qiagen |
| SDE2-1 | AAACGGCAATGGCCTACTAAA | Qiagen |
| SDE2-3 | CTACGCGATGTCAATCATGAA | Qiagen |
| SDE2-5 | GCAGTTATTGATAAGGAAAtt | Ambion |
| SDE2-7 | GAATAAGGATAAAGAGACAtt | Ambion |
| CDT2 | AAGAATTATACTGCTTATCGA | Qiagen |
| RAD18 | AAACTCAGTGTCCAACTTGCT | Qiagen |
| USP1 | TCGGCAATACTTGCTATCTTA | Qiagen |
| BRCA2 | TTGAAGAATGCAGGTTTAATA | Qiagen |
| MUS81 | CAGCCCUGGUGGAUCGAUATT | Qiagen |

**S1 Table. List of siRNA sequences**

siRNA-resistant SDE2 cDNA was generated against SDE2-1 siRNA by changing four nucleotides in the target site (shown in lower case; 5′-AAACG**c**CA**g**TGGCC**g**AC**c**AAA-3′).
