# Supplementary figures and images for: PCNA-Dependent Cleavage and Degradation of SDE2 Regulates Response to Replication Stress
Source: PLoS Genet. 2016 Dec 1;12(12):e1006465. doi: 10.1371/journal.pgen.1006465 (PMC5131917; doi:10.1371/journal.pgen.1006465)

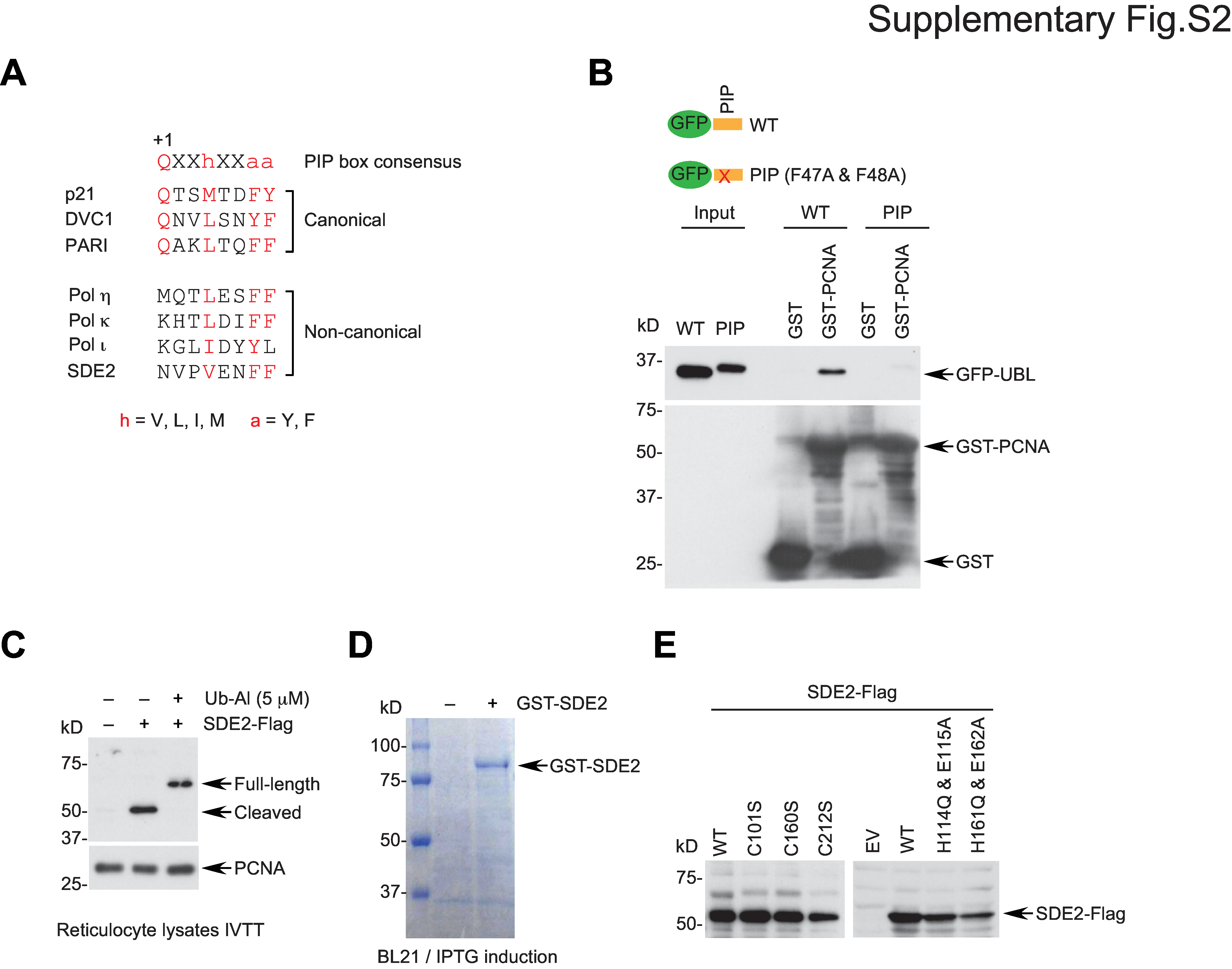

Supplement: S2 Fig — (A) Analysis of the SDE2 PIP box. Both canonical and non-canonical PIP boxes from several known PIP-box-containing proteins are presented, and conserved elements are marked in red. (B) Interaction of GFP-SDE2-UBL with PCNA. 293T cell lysates expressing GFP-SDE2-UBL wild-type or PIP mutant (F47A & F48A) were incubated with GST- or GST-PCNA-bound glutathione beads and analyzed by Western blotting. (C) SDE2-Flag proteins in vitro transcribed and translated (IVTT) from reticulocyte lysates were analyzed by Western blotting. Where indicated, 5 μM ubiquitin aldehyde (Ub-Al) was added during expression. (D) Expression of full-length GST-tagged SDE2. GST-SDE2 was induced from the E. coli BL21 strain by 0.5 mM IPTG at 30°C. Proteins were captured with glutathione-conjugated beads and visualized by Coomassie staining. (E) Conserved cysteine or histidine-glutamate residues are not required for SDE2 cleavage. The indicated SDE2-Flag wild-type or point mutants were in vitro transcribed and translated, and cleaved SDE2-Flag proteins were analyzed by Western blotting. (TIF) [file pgen.1006465.s003.tif]

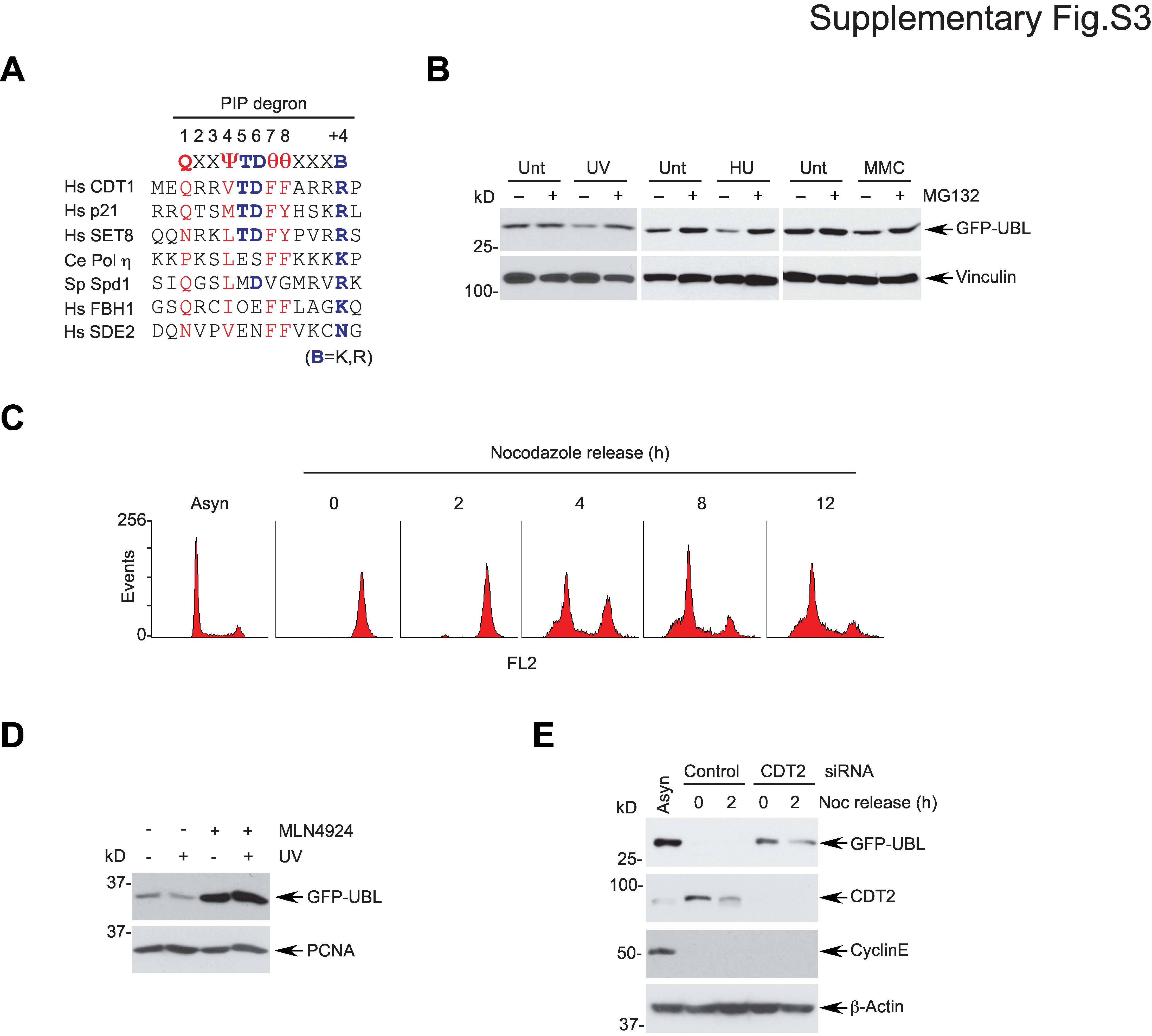

Supplement: S3 Fig — (A) Sequence alignment of PIP degron motifs present in known CDT2 substrates. Canonical PIP residues are shown in red, and PIP degron-specific residues are shown in blue. Several substrates lack elements constituting a classical PIP degron. (B) DNA-damage dependent degradation of SDE2-UBL is mediated by the proteasome. HeLa cells expressing GFP-SDE2 were left untreated (Unt) or treated with 40 J/m2 ultraviolet C (UVC) for 4 h, 2 mM hydroxyurea (HU) for 8 h, and 1 μM mitomycin C (MMC) for 16 h, and cellular GFP-UBL levels were analyzed by Western blotting. Where indicated, cells were treated with 10 μM MG132 for 4 h before harvest. (C) Cell cycle profiles of synchronized HeLa cells in Fig 3B determined by flow cytometry (D) HeLa cells expressing full-length GFP-SDE2 was treated with 1 μM MLN4924 and irradiated with 40 J/m2 UVC for 4 h. The GFP-UBL levels were analyzed by Western blotting. (E) GFP-SDE2-expressing HeLa cells transfected with siRNA control or CDT2 were synchronized by 100 ng/mL nocodazole at the G2/M phase and released for 2 h. The GFP-UBL levels were analyzed by Western blotting. (TIF) [file pgen.1006465.s004.tif]

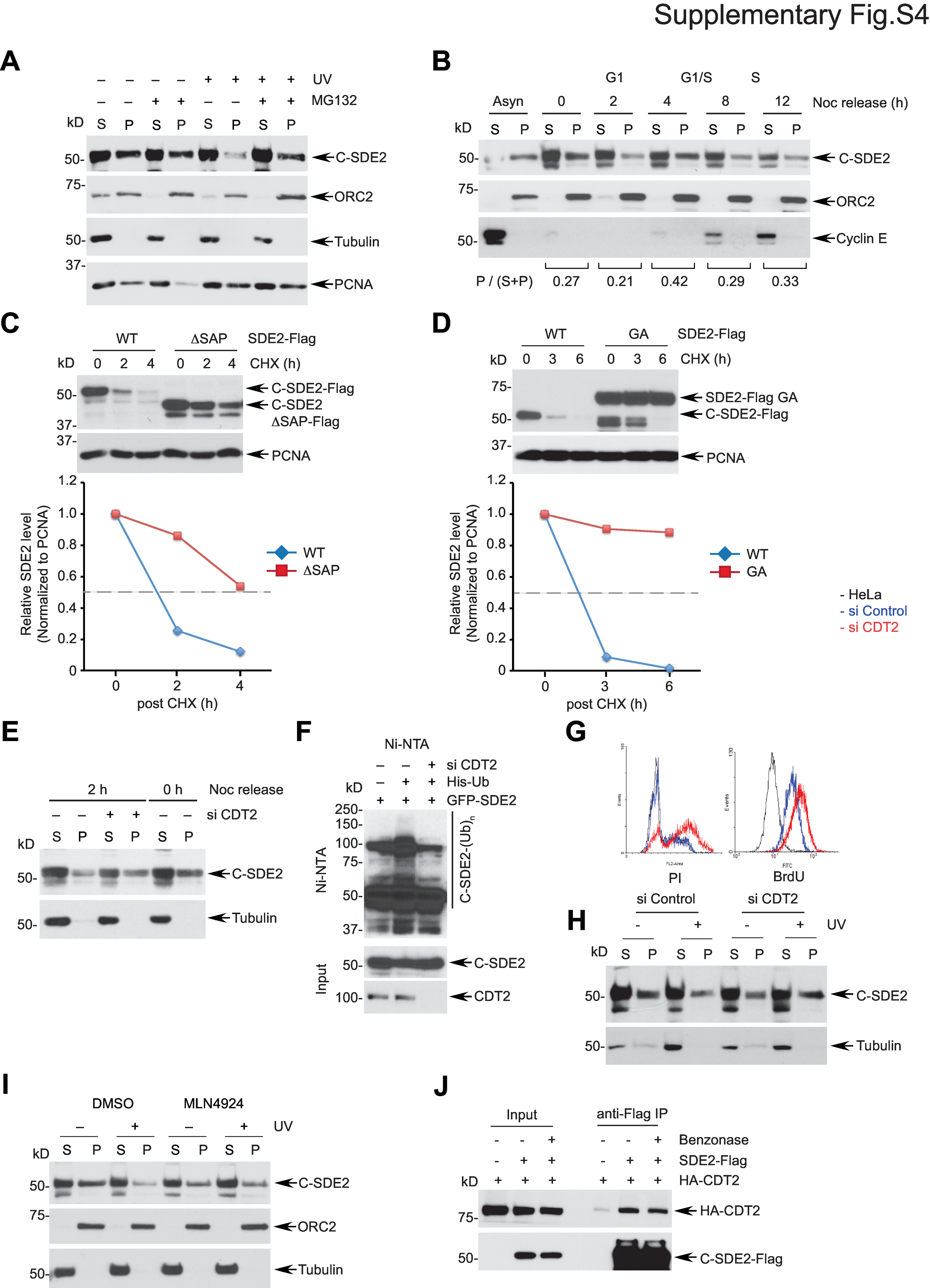

Supplement: S4 Fig — (A) Degradation of C-SDE2 is proteasome-dependent. HeLa cells were left untreated or treated with 40 J/m2 UVC for 4 h, fractionated into cytosolic/nucleoplasmic (S) and chromatin-enriched (P) fractions using CSK buffer, and the endogenous C-SDE2 levels were analyzed by Western blotting. Where indicated, cells were treated with 10 μM MG132 for 4 h before harvest. (B) C-SDE2 levels are regulated in a cell cycle-dependent manner. HeLa cells were synchronized with nocodazole for 12 h and released into fresh medium after mitotic shake-off. Cells were harvested at the indicated times, and endogenous C-SDE2 levels were analyzed by Western blotting. The cell-cycle dependent change of C-SDE2 association in chromatin is quantified by ImageJ and indicated below the blots. (C, D) The half-life of C-SDE2 is extended by ΔSAP or GA mutations. (top) HeLa cells expressing full-length SDE2-Flag wild-type or mutants were with 50 μg/mL of CHX, and cell lysates were analyzed by Western blotting. (bottom) Quantification of immunoblots by Image J. The dotted line indicates a half-life. (E) CDT2 is required for the degradation of C-SDE2 during cell cycle progression. HeLa cells transfected with siRNA control or CDT2 were synchronized with nocodazole and released, and cell lysates were analyzed by Western blotting to check endogenous C-SDE2. (F) CDT2 is required for polyubiquitination of C-SDE2. Immunoblots of the in vivo ubiquitination assay in Fig 3H were reprobed with anti-SDE2 antibody to check the polyubiquitin conjugates of C-SDE2 in the absence of CDT2. (G) Cell cycle profile and BrdU incorporation of siRNA-transfected cells were analyzed by PI staining or 30 min BrdU incubation followed by flow cytometry, respectively. (H) siRNA-transfected cells were synchronized by G2/M phase by 100 ng/mL nocodazole, treated with 40 J/m2 UVC, and released into G1 after mitotic shake-off. Degradation of C-SDE2 in chromatin was analyzed by fractionation and Western blotting. (I) Inhibition of CRL4CD [file pgen.1006465.s005.tif]

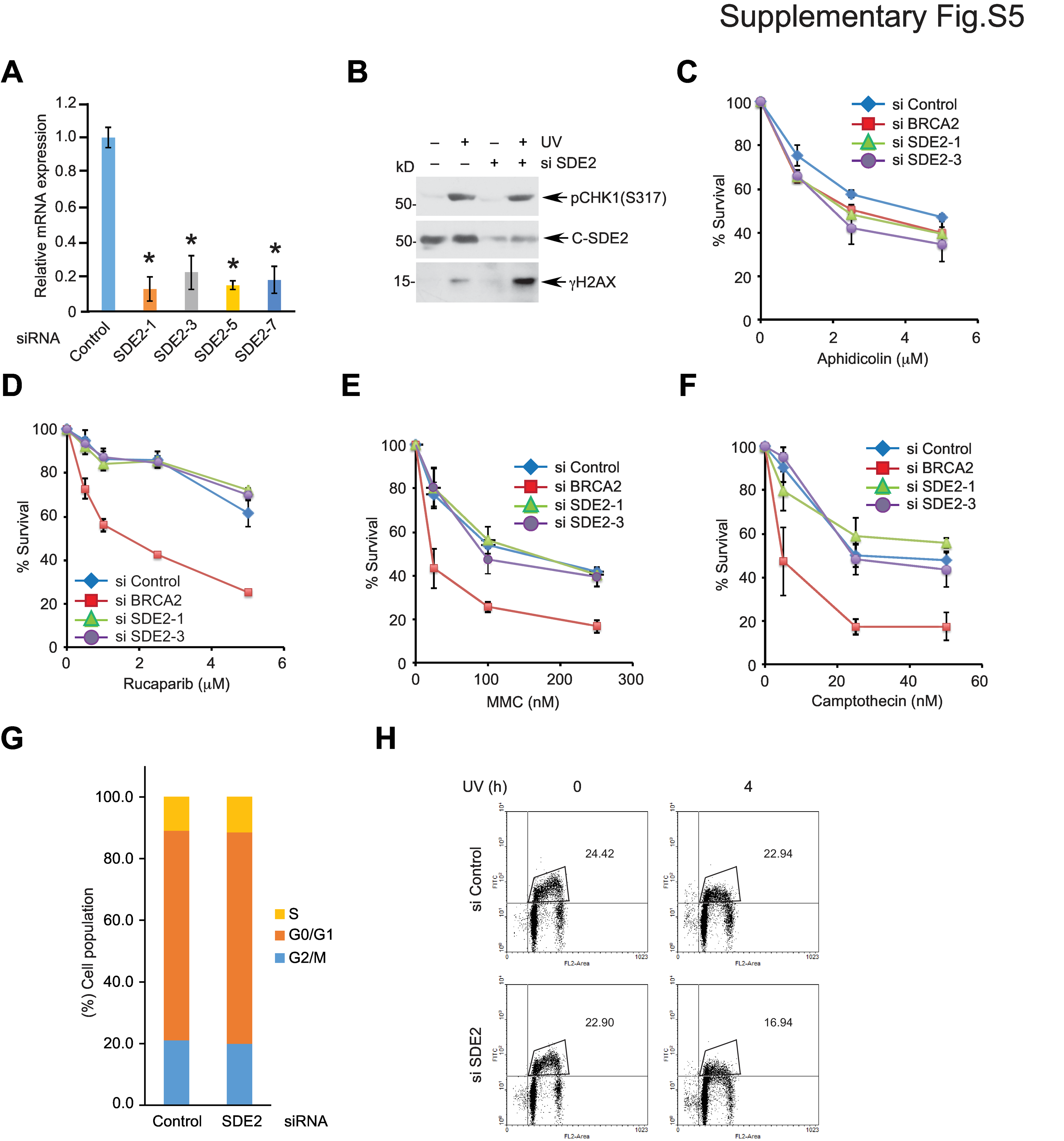

Supplement: S5 Fig — (A) Quantification of SDE2 mRNA by RT-qPCR following transfection of SDE2 siRNA for 48 h. Data shown are the mean ± SD from three independent experiments. * p < 0.01 compared with siRNA control. (B) siRNA-transfected U2OS cells were analyzed by Western blotting to confirm the elevated γH2AX levels following UVC irradiation. (C-F) Luminescence-based viability assay of HeLa cells transfected with the indicated siRNAs for 48 h. Cell viability was determined by CellTiter-Glo 5 days after treatment with the indicated doses of aphidicolin (C), rucaparib (PARP inhibitor; D), MMC (E), or camptothecin (F). (G) Cell cycle distribution of HeLa cells transfected with siRNA control or SDE2 analyzed by PI staining and flow cytometry. (H) Representative flow cytometry of cells in Fig 5H following 40 J/m2 UVC irradiation and 0.5 h BrdU pulse before harvest at 4 h post treatment. The percentage of BrdU+ cells were gated and labeled. (TIF) [file pgen.1006465.s006.tif]

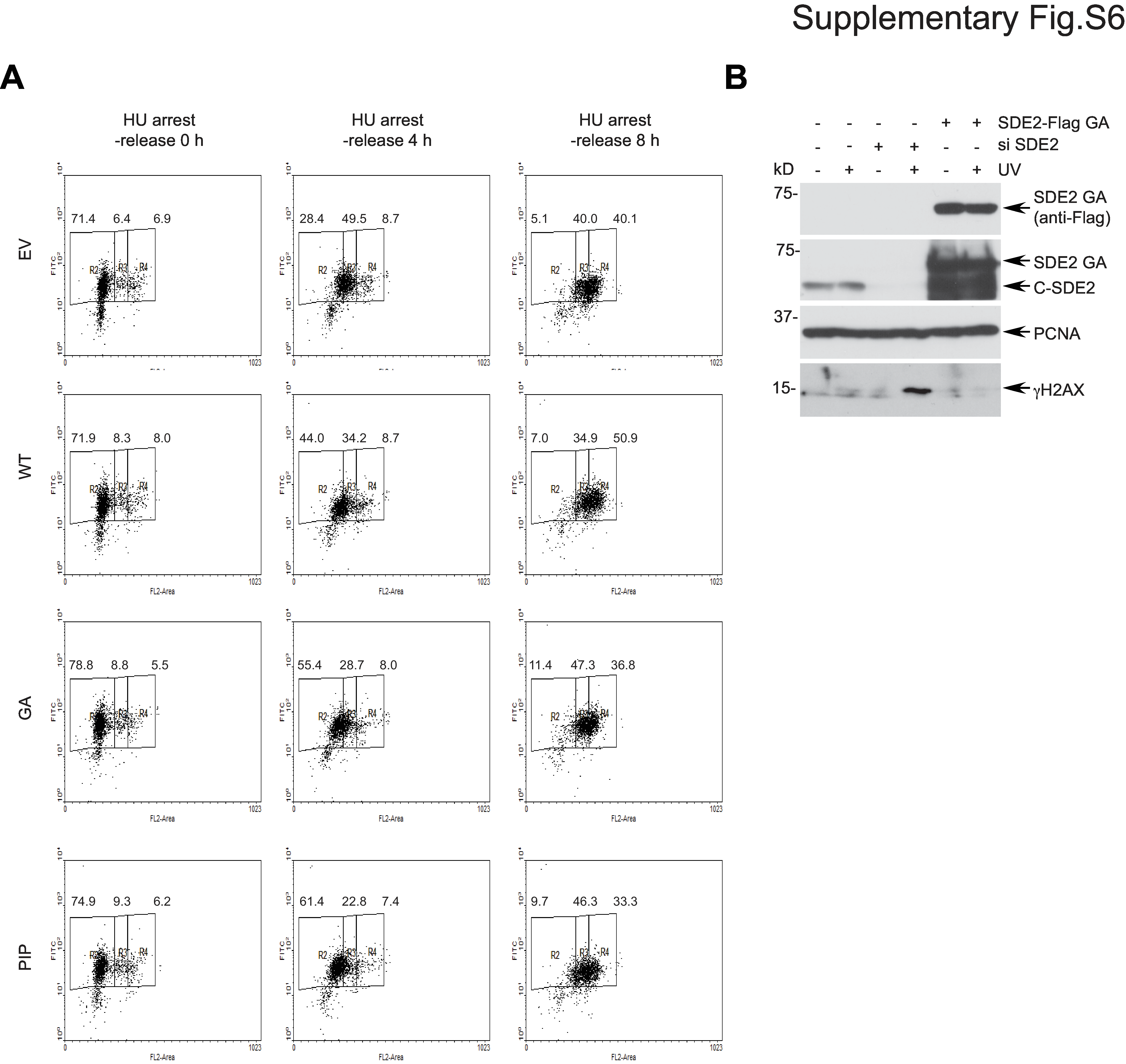

Supplement: S6 Fig — (A) A representative set of flow cytometry panels showing delayed S progression of the SDE2 GA and PIP mutants following release from HU treatment. (B) HeLa cells transfected with siRNA SDE2 or plasmid encoding the C-terminally Flag-tagged SDE2 GA mutant were irradiated with 40 J/m2 UVC for 4 h and analyzed by Western blotting. (TIF) [file pgen.1006465.s007.tif]

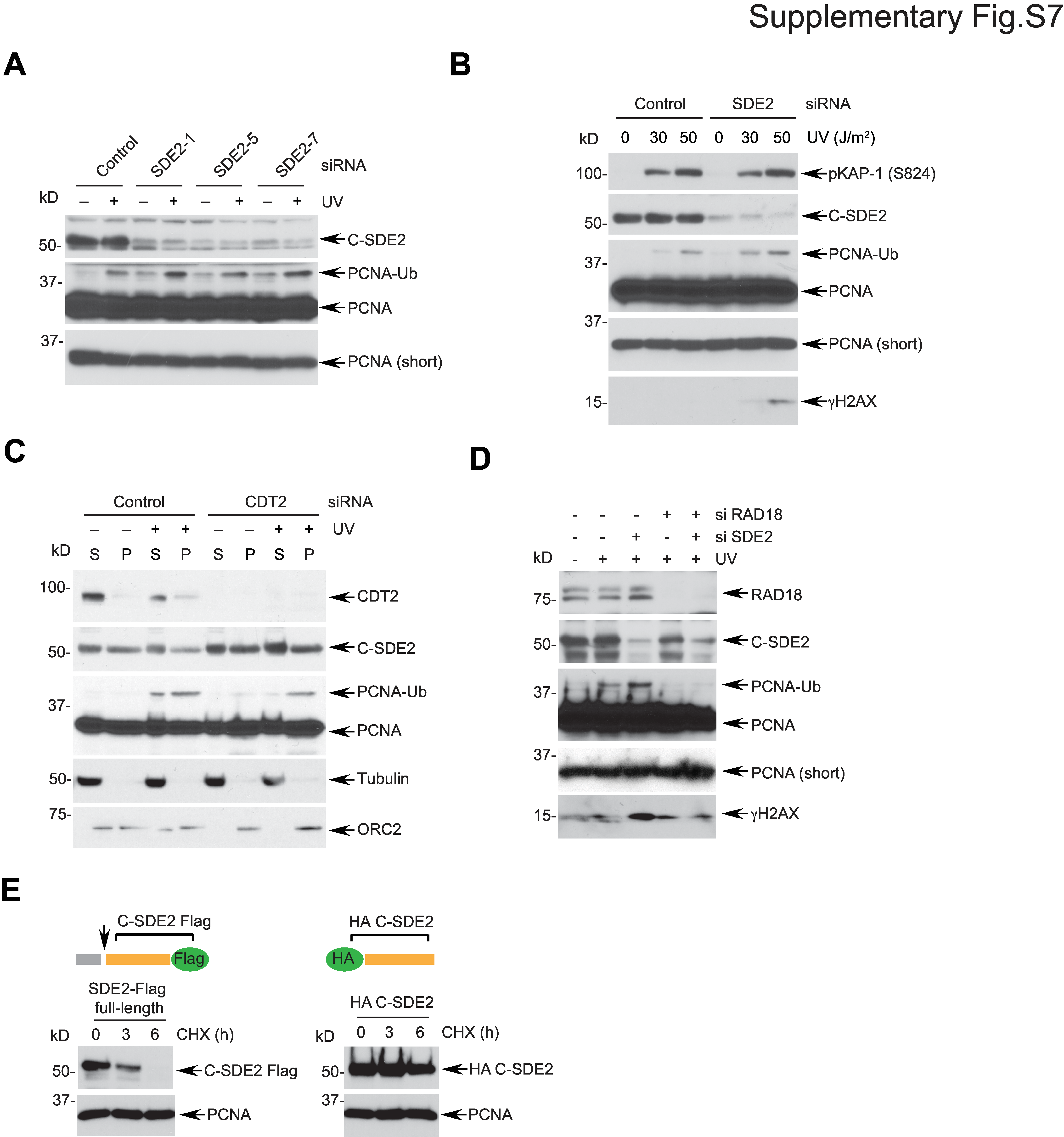

Supplement: S7 Fig — (A) Knockdown of SDE2 leads to increased PCNA monoubiquitination induced by UVC irradiation. U2OS cells transfected with the indicated siRNAs were irradiated with 40 J/m2 UVC for 4 h, and cell lysates were analyzed by Western blotting. (B) Knockdown of SDE2 increases PCNA monoubiquitination in a dose-dependent manner. siRNA-transfected HeLa cells were irradiated with the indicated doses of UVC and analyzed by Western blotting. (C) Depletion of CDT2, which prevents UVC-inducible SDE2 degradation, leads to decreased PCNA monoubiquitination. HeLa cells transfected with the indicated siRNAs were irradiated with 40 J/m2 UVC for 4 h and fractionated with CSK buffer for Western blot analysis. (D) siRNA-transfected HeLa cells were treated with 40 J/m2 UVC for 4 h, and γH2AX and PCNA-Ub levels were analyzed by Western blotting. (E) HeLa cells expressing wild-type SDE2-Flag or HA-tagged C-SDE2 were treated with 50 μg/mL of CHX, and cell lysates were analyzed by Western blotting. (TIF) [file pgen.1006465.s008.tif]

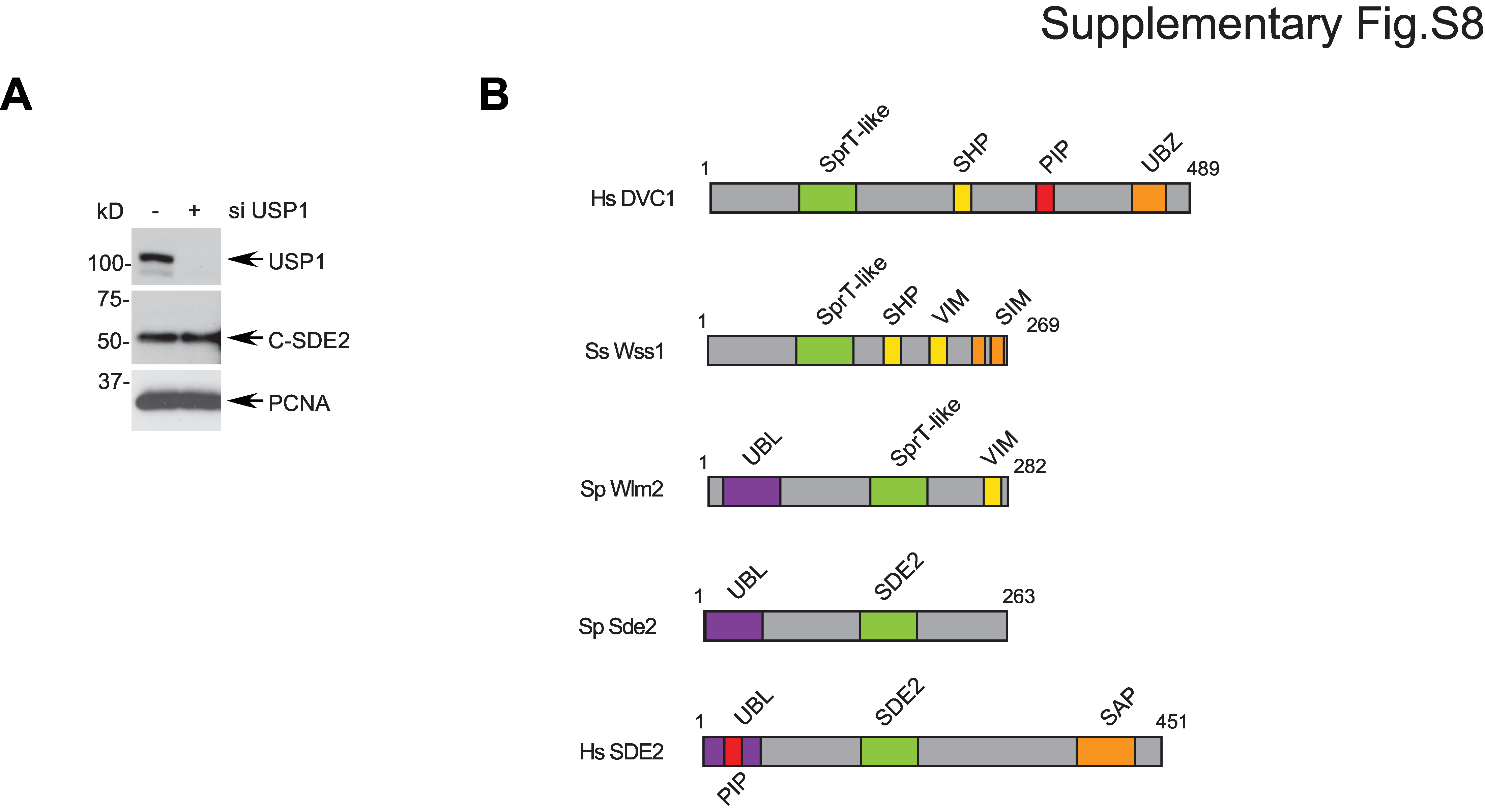

Supplement: S8 Fig — (A) HeLa cells were transfected with siRNA control or USP1, and endogenous SDE2 was analyzed by Western blotting. (B) Human DVC1 (DNA-damage protein targeting VCP), yeast Wss1 (weak suppressor of smt3), and yeast Wlm2 (Wss1-like metalloproteases) share several functional motifs including the SprT-like metalloprotease motif, Cdc48/p97-interacting motif (SHP-box and VIM), and ubiquitin- (UBZ), SUMO- (SIM) binding motif. DVC1 has a PIP box that allows for targeting to PCNA along with a UBZ motif. The N-terminus of yeast Wlm2 constitutes a ubiquitin-like domain similar to that of SDE2. hsDVC1 (Homo Sapiens NP_114407); ssWss1 (Saccharomyces Cerevisiae NP_012002.1); spWlm2 (Saccharomyces Pombe NP_588321.1); spSde2 (Saccharomyces Pombe NP_594019.1); hsSDE2 (Homo Sapiens NP_689821). (TIF) [file pgen.1006465.s009.tif]
